# Nonlinear association between stress hyperglycemia ratio and mortality in AHRF: validation through consensus clustering and supervised machine learning models

**DOI:** 10.3389/fendo.2026.1810713

**Published:** 2026-09-01

**Authors:** Qi Zhang, Tao Wang, Haoyue Wang, Liye Ji, Yang Zhao, Xiaoyong Geng, Zhiyong Wang, Yaoyao Tang, Mingxing Fang

**Affiliations:** 1 Department of Critical Care Medicine, Hebei Medical University Third Hospital, Shijiazhuang, China; 2 Critical Disease Data Analysis and Intelligent Diagnosis and Treatment Engineering Research Center of Hebei Province, Hebei Medical University Third Hospital, Shijiazhuang, China; 3 Department of Medical Imaging, The People’s Hospital of Guizhou, Guiyang, China; 4 Applied Statistics, College of Science, Hebei University of Technology, Tianjin, China; 5 Department of Endocrinology, Shijiazhuang Traditional Chinese Hospital, Shijiazhuang, China; 6 Department of Cardiology, Hebei Medical University Third Hospital, Shijiazhuang, China

**Keywords:** acute hypoxemic respiratory failure, cluster analysis, machine learning, mortality, stress hyperglycemia ratio

## Abstract

**Purpose:**

Acute hypoxemic respiratory failure (AHRF), a life-threatening condition in critically ill patients, is associated with poor clinical outcomes. Although the stress hyperglycemia ratio (SHR) may reflect acute metabolic stress, its prognostic role in AHRF remains unclear. This study investigated the association between SHR and mortality risk in patients with AHRF.

**Methods:**

We retrospectively analyzed 2514 patients with AHRF from the MIMIC-IV database. Restricted cubic spline analysis was used to assess the potential non-linear association between SHR and 28-day all-cause mortality. Kaplan-Meier survival analysis and Cox proportional hazards models were used to evaluate mortality risk. Secondary predictive analyses assessed the incremental value of SHR beyond SOFA and evaluated 101 candidate machine-learning models or model combinations. Consensus clustering was used as an exploratory approach to identify data-driven clinical phenotypes.

**Results:**

In the MIMIC-IV cohort, 28-day all-cause mortality increased most clearly among patients with elevated SHR. In the primary parsimonious Cox model, the highest SHR quartile was associated with increased 28-day all-cause mortality compared with the lowest quartile (HR = 2.972, 95% CI: 2.108-4.189, P < 0.001). Restricted cubic spline analysis suggested a possible non-linear association, with a data-derived inflection point around 0.929 in this cohort. Event-distribution and extreme-value sensitivity analyses indicated that the association between elevated SHR and mortality was not driven solely by extreme SHR values. In secondary predictive analyses, SHR improved discrimination beyond SOFA, and the final CoxBoost + SuperPC model achieved an optimism-corrected C-index of 0.803 in the training cohort and a C-index of 0.826 in the independent validation cohort. Consensus clustering identified three exploratory clinical phenotypes with different severity profiles and mortality risks. Phenotype-stratified analyses suggested a clearer SHR-mortality association in Phenotype II, although formal interaction testing with dichotomized SHR was not statistically significant.

**Conclusion:**

SHR was associated with 28-day all-cause mortality in patients with AHRF and may serve as a potential prognostic marker for risk stratification. The most consistent evidence supported an increased mortality risk among patients with elevated SHR, whereas the possible nonlinear pattern, particularly in the lower SHR range, requires further validation. Exploratory phenotype-specific findings should be interpreted cautiously and validated in independent cohorts.

## Background

Acute hypoxemic respiratory failure (AHRF) is a severe complication observed in critically ill patients and was operationally defined in this study as a PaO2/FiO2 ratio of ≤300 mmHg. AHRF can result from reduced inspired oxygen pressure, impaired alveolar oxygen diffusion, inadequate alveolar ventilation, intrapulmonary shunting, and ventilation-perfusion mismatch (1, 2). Patients with AHRF frequently exhibit severe oxygenation impairment and multi-organ dysfunction, contributing to persistently high mortality. Despite advances in intensive care and mechanical ventilation strategies, AHRF remains an important global public health concern.

Stress hyperglycemia is an acute hyperglycemic response related to physiological or pathological stress and may be associated with endocrine, immune, and metabolic dysregulation (3). Previous studies have suggested that hyperglycemia may be related to an impaired immune response and increased susceptibility to infection (4). To more accurately assess acute hyperglycemia relative to chronic glycemic background, researchers have proposed the stress hyperglycemia ratio (SHR), defined as admission blood glucose divided by estimated average glucose derived from HbA1c (5). SHR may reflect acute metabolic stress in critically ill patients and has been reported to have prognostic value in several critical illness settings (6). In this study, we evaluated the association between SHR and clinical outcomes in patients with AHRF and explored whether SHR could contribute to risk stratification.

## Methods

### Study design and population

This study was conducted based on the MIMIC-IV database, which includes information on patients treated in the intensive care unit of Beth Israel Deaconess Medical Center between 2008 and 2019. One of the authors completed the required courses and assessments of the Collaborative Institutional Training Initiative (CITI) for ‘Data or Specimen Studies’ and was granted access to the MIMIC database (Certification Number: 9535772). As all patient data in the database are fully anonymized, ethical approval was not required for this study. AHRF was operationally defined as a PaO2/FiO2 ratio of ≤300 mmHg. The PaO2/FiO2 ratio was extracted from the bg table, which contains blood gas-derived variables in the MIMIC-IV database. Patients were considered eligible at the first time point when they met this predefined PaO2/FiO2 criterion. For patients who met the criterion during multiple hospital or ICU admissions, only the first qualifying admission was included. Adult patients aged >18 years with an ICU length of stay longer than 24 hours were included. Exclusion criteria included (1): patients with missing glycated hemoglobin (HbA1c) data within 48 hours before or after the diagnosis (2); patients without recorded blood glucose levels within 24 hours after the diagnosis; and (3) repeated ICU admissions after the first qualifying ICU admission.

### Data extraction

Data were extracted from the MIMIC-IV database using Structured Query Language (SQL). The collected variables included (1): demographic characteristics: age, gender, height, weight, and body mass index (BMI) (2); severity scores: Sequential Organ Failure Assessment (SOFA) score and Simplified Acute Physiology Score II (SAPS II) (3); vital signs: heart rate, respiratory rate, systolic blood pressure (SBP), mean arterial pressure (MAP), and diastolic blood pressure (DBP) (4); laboratory events: white blood cell count (WBC), red blood cell count (RBC), red cell distribution width (RDW), platelet count (PLT), blood urea nitrogen (BUN), creatinine, anion gap, bicarbonate, calcium, lactate, international normalized ratio (INR), prothrombin time (PT), partial thromboplastin time (PTT), total bilirubin, ALT, AST, alkaline phosphatase (ALP), fibrinogen, hemoglobin, sodium, potassium, serum glucose, and HbA1c (5); blood gas analysis parameters: arterial pH, PaO2, PaCO2, and SpO2 (6); comorbidities and treatments: glucocorticoid use, hypertension, pneumonia, septic shock, stroke, type 1 diabetes, type 2 diabetes, renal disease, malignant cancer, and congestive heart failure; and (7) outcomes: 28-day all-cause mortality, 60-day all-cause mortality, 90-day all-cause mortality, in-hospital all-cause mortality, and ICU length of stay. SHR was calculated as serum glucose (mg/dL) divided by the estimated average glucose derived from HbA1c, using the formula: SHR = serum glucose/(28.7 × HbA1c (%) - 46.7). The SHR concept was based on Roberts et al., and the estimated-average-glucose equation was derived from the ADAG study by Nathan et al. (5, 7).

### Data cleaning and processing

Data cleaning and preprocessing were performed after the construction of the patient-level analytic dataset. For variables with multiple measurements within the first 24 hours after cohort entry, patient-level summary values were first calculated. In the primary analysis, outliers were handled using the 1.5 × interquartile range (IQR) rule before imputation. Given that extreme physiological and laboratory values in critically ill patients may represent true disease severity rather than erroneous measurements, two additional outlier-handling strategies were evaluated in sensitivity analyses. First, only clinically implausible or physiologically impossible values were removed based on clinical plausibility. Second, winsorization was applied to limit the influence of extreme values while retaining patients with clinically meaningful extreme measurements. Variables with more than 20% missingness were excluded to reduce imputation-related uncertainty. The remaining variables with missingness ≤20% were imputed using the MissForest algorithm. Outcome variables, including 28-day all-cause mortality and survival time, were not included in the imputation model to avoid outcome leakage. Sensitivity analyses were performed using multiple imputation by chained equations (MICE).

### Statistical analysis

Data were analyzed using R version 4.3.1. Normally distributed continuous variables are expressed as the mean ± standard deviation (mean ± SD), with group comparisons performed using the independent t-test. For continuous variables non-normally distributed, values are presented as the median (interquartile range) (Median (IQR)), and comparisons between groups are made using the Wilcoxon rank-sum test. Categorical variables are reported as percentages, with between-group comparisons conducted using the Chi-square test or Fisher’s exact test, as appropriate. Survival curves were generated using the Kaplan-Meier method, with between-group survival differences assessed by the log-rank test. The impact of SHR on the prognosis of AHRF patients was evaluated using both logistic regression and Cox proportional hazards regression models. Kaplan Meier survival curves were used to compare the 28-day survival rates among the groups according to the log-rank test. A two-tailed *P* value of < 0.05 was considered statistically significant.

### Restricted cubic splines

Restricted cubic spline (RCS) analysis was used to assess the potential non-linear relationship between SHR and 28-day all-cause mortality. Between 3 and 7 knots were evaluated, with knot positions determined based on percentiles of SHR and model selection based on the lowest Akaike Information Criterion (AIC). Model 1 was unadjusted. Model 2 was defined as the primary parsimonious model and adjusted for age, gender, BMI, SOFA score, type 2 diabetes, hypertension, pneumonia, septic shock, stroke, renal disease, malignant cancer, congestive heart failure, and glucocorticoid use. Acute physiological and laboratory variables, including lactate, bicarbonate, anion gap, blood gas parameters, coagulation markers, and hematologic markers, were not included in the primary model because they may represent downstream manifestations of acute illness or overlap with global disease severity. The estimated inflection point was interpreted as data-derived and exploratory rather than as an established clinical cutoff.

### Feature selection

The Boruta algorithm (8) was used as an exploratory feature-importance screen based on random forest modeling. Shadow features were generated by randomly permuting copies of the original variables, and original variables were compared with these shadow features across repeated iterations. Because the final predictive workflow used CoxBoost for feature selection followed by SuperPC for survival prediction, Boruta results were interpreted as descriptive feature-importance findings rather than as the basis for causal inference.

### Machine learning model development

Machine learning analysis was performed as a secondary predictive analysis to evaluate whether SHR and routinely available clinical variables could contribute to 28-day all-cause mortality prediction beyond conventional regression analyses. To assess the incremental predictive value of SHR beyond established severity scoring, three logistic regression models were compared for the binary 28-day all-cause mortality endpoint: SOFA alone, SHR alone, and SOFA + SHR. Model performance was evaluated using the C-index/AUC, 95% confidence intervals for AUC, calibration intercept, calibration slope, and Brier score, and AUCs were compared using DeLong tests. A total of 101 candidate machine-learning models or two-step model combinations were evaluated. For the final CoxBoost + SuperPC model, CoxBoost was used for feature selection, and SuperPC was used for final survival prediction. The CoxBoost penalty parameter was optimized using optimCoxBoostPenalty with start.penalty = 500, and the optimal number of boosting steps was selected by 10-fold cross-validation using cv.CoxBoost with maxstepno = 500, K = 10, and type = ‘verweij’. SuperPC was trained with type = ‘survival’ and s0.perc = 0.5, and the threshold was selected by 10-fold cross-validation with n.threshold = 20, n.fold = 10, n.components = 3, and min.features = 5. Bootstrap optimism correction was performed in the training cohort by repeating the full modeling procedure, and the final locked model was evaluated in an independent validation cohort that was not used for feature selection, model training, hyperparameter tuning, or cutoff determination. Pairwise comparisons between CoxBoost + SuperPC and top-performing candidate models were performed using 28-day time-dependent AUC comparison, with DeLong tests added as supplementary analyses for the binary 28-day endpoint.

### Cluster analysis

The optimal number of clusters and the distribution of K-means clustering under resampling conditions were evaluated through consensus clustering (CC), employing a subsampling ratio of 80%, 1000 iterations, and exploring cluster numbers (k) from 2 to 6. The ideal number of clusters was selected based on the heatmap of the Consistency Matrix (CM), the Cumulative Distribution Function (CDF), and intra-cluster consistency scores. A score near 1 indicated greater cluster stability. Simultaneously, the proportion of ambiguously clustered pairs (PAC) was employed to automatically determine the optimal number of clusters, with a PAC value close to 0 indicating better clustering stability. After determining the optimal cluster, results were visualized using t-distributed stochastic neighbor embedding (t-SNE). The relationship between the clinical features of different phenotypes and mortality was analyzed, with clinical features presented as percentages or mean ± standard deviation. Group comparisons were made using the chi-square test, analysis of variance (ANOVA), and Wilcoxon’s test. All statistical analyses were performed using R version 4.3.1. 

## Results

### Participant characteristics and clinical outcomes

**Figure 1:**
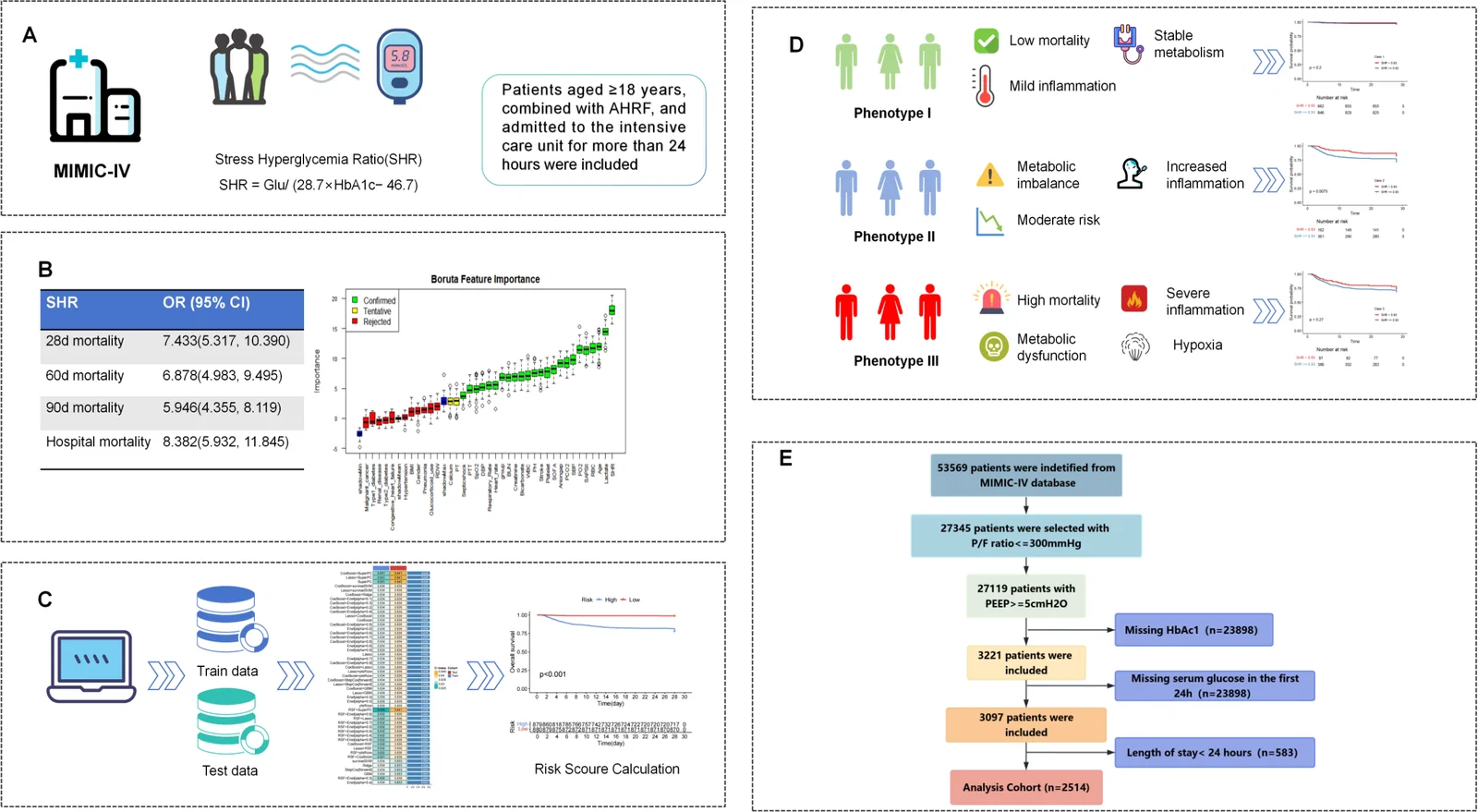
Study design and analytical workflow. **(A)** Definition of the study population and calculation of the stress hyperglycemia ratio (SHR). **(B)** Associations between SHR and prespecified mortality outcomes and Boruta feature-importance analysis. **(C)** Training and validation workflow for the predictive model and risk-score calculation. **(D)** Clinical characteristics and survival patterns of the three consensus-clustering phenotypes. **(E)** Flowchart of patient selection and cohort construction.

**Figure 2:**
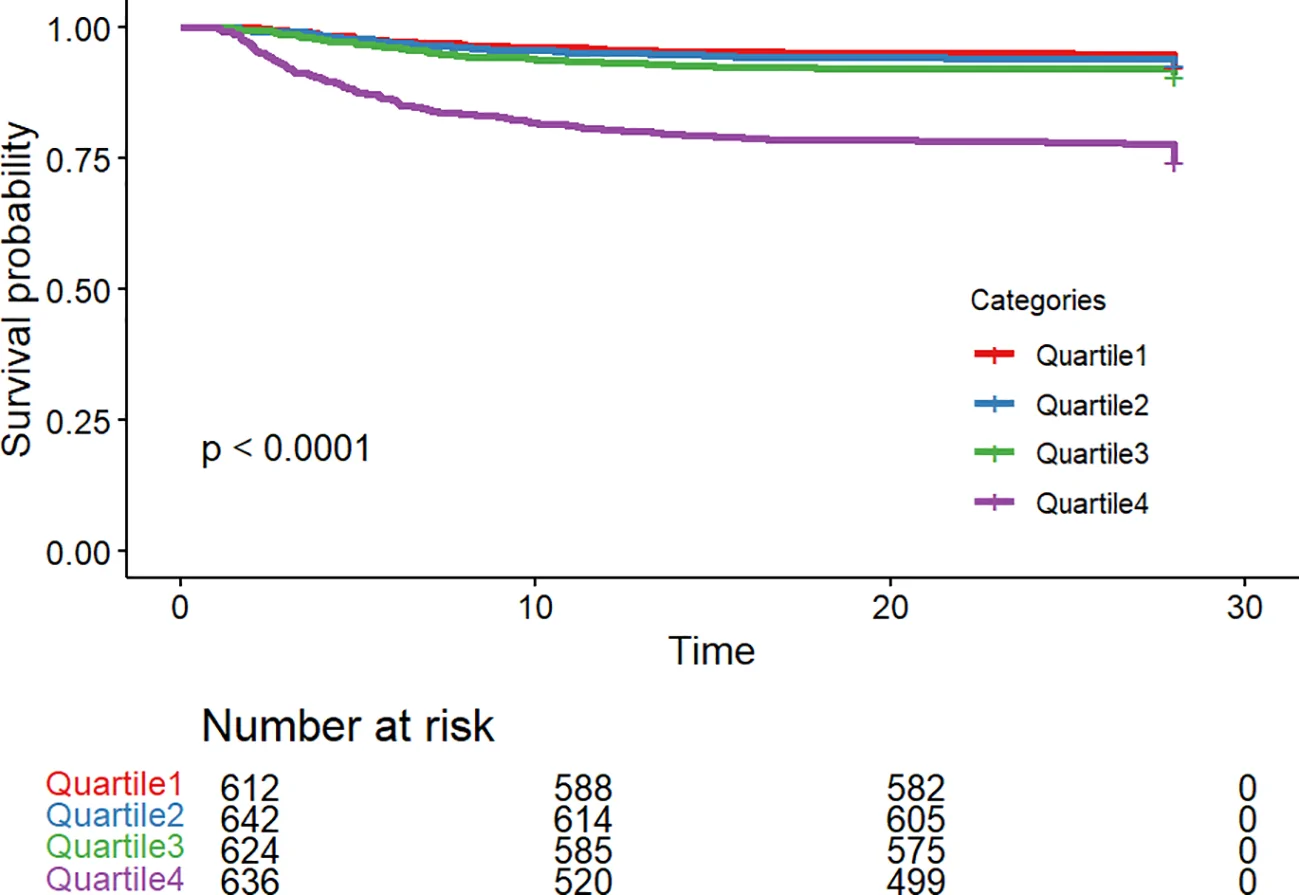
28-day KM survival curve based on SHR categories. Quartile 1 (0.19 ≤ SHR < 0.84), Quartile 2 (0.84 ≤ SHR < 1.02), Quartile 3 (1.02 ≤ SHR < 1.20), and Quartile 4 (1.20 ≤ SHR ≤ 4.42).

A total of 2,514 patients diagnosed with AHRF were included in the analysis (**Figure 1**). The associations between SHR and prespecified mortality outcomes are summarized in **Table 1**. The proportion of missing data for each variable is illustrated in **Supplementary Figure 1**, and missingness details are provided in **Supplementary Table 4**. Patients were categorized into four quartiles based on SHR: Quartile 1 (0.19 ≤ SHR < 0.84), Quartile 2 (0.84 ≤ SHR < 1.02), Quartile 3 (1.02 ≤ SHR < 1.20), and Quartile 4 (1.20 ≤ SHR ≤ 4.42). Compared with other quartiles, patients in Quartile 4 had higher SOFA scores, BUN, creatinine, lactate, white blood cell count, anion gap, and glucocorticoid use, as well as lower bicarbonate, serum calcium, and PaO2 (**Table 2**). In Cox regression analysis, the association between SHR and 28-day all-cause mortality remained consistent across the revised adjustment framework. In the primary parsimonious model, Quartile 4 was associated with increased 28-day all-cause mortality compared with Quartile 1 (HR = 2.972, 95% CI: 2.108-4.189, P < 0.001; **Table 3**). Kaplan-Meier survival analysis further showed that patients in Quartile 4 had the lowest 28-day survival probability (**Figure 2**).

**Table 1:** Associations between SHR and prespecified mortality outcomes.

SHR	OR	Lower bound 95% CI	Upper bound 95% CI	*P*-value
28-day all-cause mortality	7.433	5.317	10.39	<0.001
60-day all-cause mortality	6.878	4.983	9.495	<0.001
90-day all-cause mortality	5.946	4.355	8.119	<0.001
In-hospital all-cause mortality	8.382	5.932	11.845	<0.001

**Table 2:** Patient baseline demographic and clinical characteristics by SHR.

Characteristic	Overall N = 2,514	Quartile 1 N = 612	Quartile 2 N = 642	Quartile 3 N = 624	Quartile 4 N = 636	*P-*value
Age	68.4 (59.9, 77.0)	69.1 (59.4, 77.2)	69.2 (61.5, 77.5)	68.9 (60.3, 76.4)	66.6 (57.5, 76.6)	0.004
SHR	1.0 (0.8, 1.2)	0.7 (0.6, 0.8)	0.9 (0.9, 1.0)	1.1 (1.1, 1.1)	1.4 (1.3, 1.6)	<0.001
Gender						>0.900
Male	1,655 (66%)	403 (66%)	425 (66%)	415 (67%)	412 (65%)	
Female	859 (34%)	209 (34%)	217 (34%)	209 (33%)	224 (35%)	
BMI	29.1 (25.8, 33.4)	29.6 (26.1, 33.7)	29.4 (26.2, 33.6)	28.9 (25.9, 32.9)	28.4 (24.9, 33.0)	<0.001
SOFA	6.0 (5.0, 9.0)	6.0 (5.0, 8.0)	6.0 (5.0, 8.0)	6.0 (5.0, 8.0)	7.0 (5.0, 10.0)	<0.001
Heart rate	82.5 (75.2, 90.9)	83.2 (76.1, 90.9)	81.7 (74.5, 89.3)	82.0 (74.2, 90.1)	84.0 (75.8, 93.8)	<0.001
BUN	16.5 (12.5, 23.5)	16.4 (12.5, 24.0)	16.0 (12.0, 21.0)	16.0 (12.8, 22.0)	18.5 (13.5, 27.1)	<0.001
Creatinine	1.0 (0.8, 1.3)	1.0 (0.8, 1.3)	0.9 (0.8, 1.2)	1.0 (0.8, 1.2)	1.0 (0.8, 1.6)	<0.001
Aniongap	12.5 (11.0, 14.8)	12.0 (10.0, 14.0)	12.0 (10.5, 14.0)	12.8 (11.0, 14.5)	13.7 (12.0, 16.0)	<0.001
Bicarbonate	23.0 (21.0, 25.0)	23.0 (21.5, 25.0)	23.0 (21.0, 25.0)	23.0 (21.0, 24.2)	22.0 (19.7, 24.5)	<0.001
Lactate	1.9 (1.5, 2.5)	1.8 (1.4, 2.3)	1.8 (1.5, 2.3)	1.8 (1.4, 2.3)	2.1 (1.6, 2.8)	<0.001
Calcium	1.2 (1.1, 1.2)	1.2 (1.1, 1.2)	1.2 (1.1, 1.2)	1.2 (1.1, 1.2)	1.1 (1.1, 1.2)	<0.001
SBP	112.5 (106.0, 119.8)	112.5 (106.5, 119.1)	112.2 (106.2, 119.2)	112.4 (105.4, 120.1)	113.0 (105.6, 122.4)	0.700
DBP	59.0 (53.9, 64.3)	58.1 (52.9, 62.7)	58.6 (54.0, 63.7)	59.2 (54.2, 64.5)	60.3 (54.5, 66.3)	<0.001
Respiratory Rate	18.9 (17.1, 20.8)	18.6 (17.0, 20.4)	18.8 (17.0, 20.5)	18.9 (16.9, 20.7)	19.4 (17.4, 21.7)	<0.001
pH	7.4 (7.4, 7.4)	7.4 (7.4, 7.4)	7.4 (7.4, 7.4)	7.4 (7.4, 7.4)	7.4 (7.3, 7.4)	<0.001
PaO2	183.7 (134.0, 229.4)	194.8 (147.4, 231.7)	189.4 (140.5, 236.0)	184.6 (134.5, 234.5)	163.0 (117.8, 210.8)	<0.001
PaCO2	40.0 (37.3, 43.0)	40.3 (37.5, 43.1)	40.3 (37.9, 43.0)	40.0 (37.3, 42.6)	39.6 (36.5, 43.0)	0.017
SpO2	96.5 (95.8, 97.3)	96.5 (95.9, 97.2)	96.5 (95.8, 97.0)	96.5 (95.9, 97.2)	96.5 (95.6, 97.5)	0.800
PTT	31.5 (28.2, 36.2)	31.8 (28.3, 36.6)	31.2 (28.0, 35.0)	31.2 (27.8, 35.9)	32.0 (28.5, 37.4)	0.012
PT	14.0 (13.0, 15.1)	13.9 (13.0, 15.1)	14.0 (13.1, 15.1)	13.9 (12.9, 15.1)	14.1 (13.0, 15.2)	0.400
WBC	12.7 (9.9, 15.7)	12.2 (9.7, 15.4)	12.3 (9.6, 15.5)	12.8 (10.0, 15.5)	13.3 (10.2, 16.7)	<0.001
RBC	3.5 (3.1, 3.9)	3.5 (3.1, 3.9)	3.5 (3.2, 3.9)	3.5 (3.1, 3.9)	3.5 (3.1, 4.0)	0.300
RDW	14.0 (13.2, 14.9)	14.0 (13.3, 14.8)	13.9 (13.2, 14.8)	13.9 (13.2, 14.8)	14.1 (13.2, 15.0)	0.200
Platelet	160.7 (126.8, 205.5)	166.7 (131.3, 210.3)	157.6 (128.0, 195.0)	157.9 (124.7, 200.0)	163.3 (122.2, 212.3)	0.034
Glucocorticoid use						0.058
No	2,427 (97%)	590 (96%)	625 (97%)	608 (97%)	604 (95%)	
Yes	87 (3.5%)	22 (3.6%)	17 (2.6%)	16 (2.6%)	32 (5.0%)	
Hypertension						<0.001
No	1,500 (60%)	330 (54%)	347 (54%)	388 (62%)	435 (68%)	
Yes	1,014 (40%)	282 (46%)	295 (46%)	236 (38%)	201 (32%)	
Pneumonia						<0.001
No	1,880 (75%)	472 (77%)	505 (79%)	474 (76%)	429 (67%)	
Yes	634 (25%)	140 (23%)	137 (21%)	150 (24%)	207 (33%)	
Septic shock						<0.001
No	2,298 (91%)	565 (92%)	602 (94%)	581 (93%)	550 (86%)	
Yes	216 (8.6%)	47 (7.7%)	40 (6.2%)	43 (6.9%)	86 (14%)	
Stroke						0.300
No	2,030 (81%)	507 (83%)	519 (81%)	505 (81%)	499 (78%)	
Yes	484 (19%)	105 (17%)	123 (19%)	119 (19%)	137 (22%)	
Type 1 diabetes						0.200
No	2,489 (99%)	602 (98%)	636 (99%)	621 (100%)	630 (99%)	
Yes	25 (1.0%)	10 (1.6%)	6 (0.9%)	3 (0.5%)	6 (0.9%)	
Type 2 diabetes						<0.001
No	1,973 (78%)	377 (62%)	521 (81%)	539 (86%)	536 (84%)	
Yes	541 (22%)	235 (38%)	121 (19%)	85 (14%)	100 (16%)	
Renal disease						0.034
No	1,929 (77%)	444 (73%)	494 (77%)	493 (79%)	498 (78%)	
Yes	585 (23%)	168 (27%)	148 (23%)	131 (21%)	138 (22%)	
Malignant cancer						0.010
No	2,314 (92%)	552 (90%)	610 (95%)	571 (92%)	581 (91%)	
Yes	200 (8.0%)	60 (9.8%)	32 (5.0%)	53 (8.5%)	55 (8.6%)	
Congestive heart failure						0.057
No	1,452 (58%)	333 (54%)	395 (62%)	367 (59%)	357 (56%)	
Yes	1,062 (42%)	279 (46%)	247 (38%)	257 (41%)	279 (44%)	

**Table 3:** Cox regression model (28-day all-cause mortality).

Categories	HR	Lower bound 95% CI	Upper bound 95% CI	P-value
Model 1 - unadjusted				
Q1	Reference	Reference	Reference	Reference
Q2	0.957	0.636	1.440	0.833
Q3	1.257	0.853	1.851	0.247
Q4	3.892	2.806	5.398	<0.001
Model 2 - primary parsimonious				
Q1	Reference	Reference	Reference	Reference
Q2	0.969	0.640	1.467	0.882
Q3	1.170	0.787	1.737	0.438
Q4	2.972	2.108	4.189	<0.001
Model 3 - extended sensitivity				
Q1	Reference	Reference	Reference	Reference
Q2	0.883	0.580	1.344	0.562
Q3	1.016	0.682	1.515	0.936
Q4	2.102	1.464	3.018	<0.001

Model 1, unadjusted. Model 2, the primary parsimonious model adjusted for age, gender, BMI, SOFA score, type 2 diabetes, hypertension, pneumonia, septic shock, stroke, renal disease, malignant cancer, congestive heart failure, and glucocorticoid use. Model 3, extended sensitivity model, further adjusted for broader physiological and laboratory variables.

### The relationship between SHR levels and 28-day all-cause mortality was analyzed by restricted cubic spline

**Figure 3:**
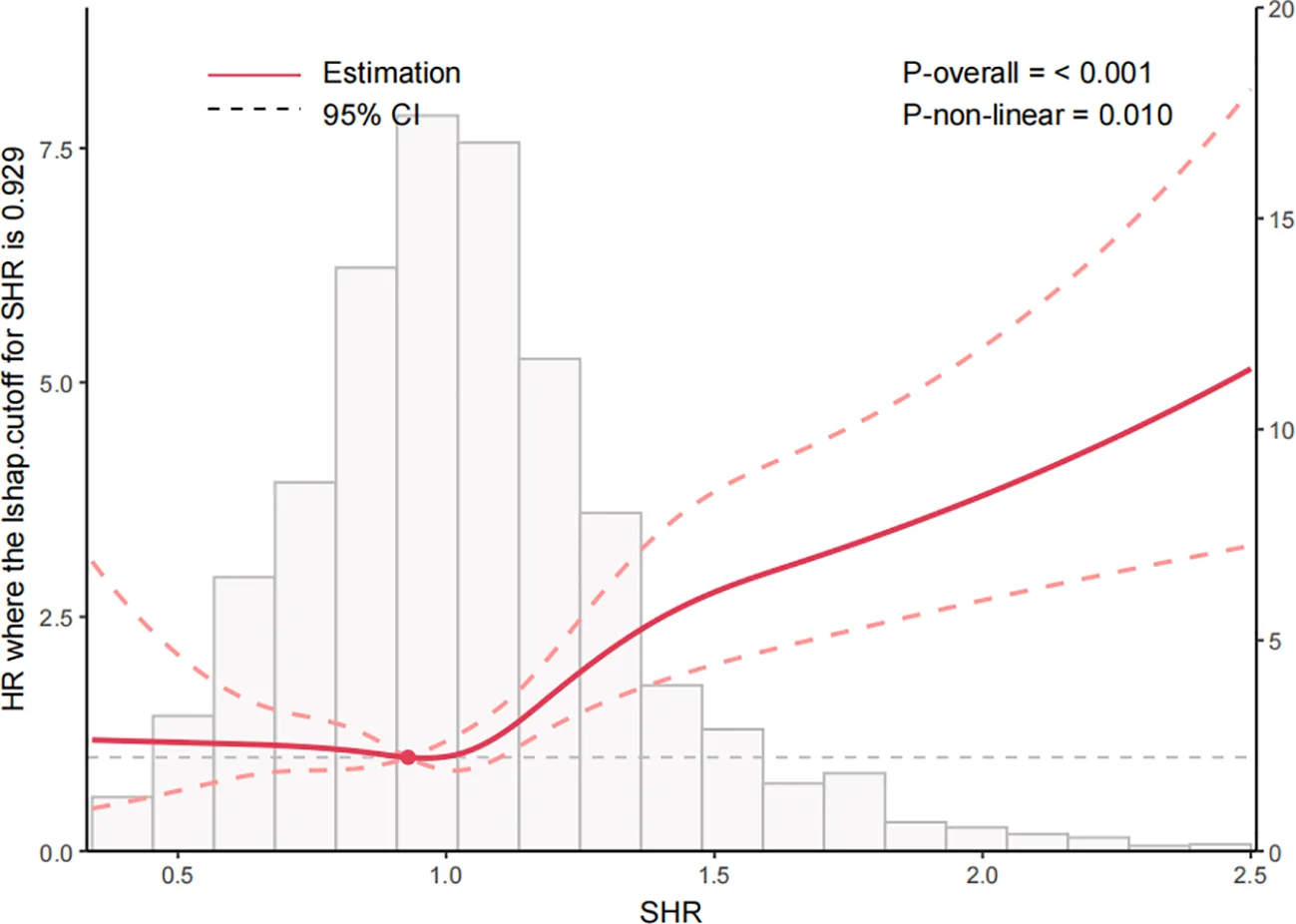
Restricted cubic spline analysis of the association between SHR and 28-day all-cause mortality. The overall P value was <0.001 and the non-linearity P value was 0.010. The estimated inflection point around SHR = 0.929 was data-derived and should be interpreted cautiously rather than as an established clinical cutoff.

Restricted cubic spline analysis suggested a possible non-linear association between SHR and 28-day all-cause mortality, with a data-derived inflection point around 0.929 in this cohort (**Figure 3**). This value should not be interpreted as an established clinical cutoff. The strongest and most consistent evidence supported increased mortality risk at higher SHR levels.

To evaluate the stability of the nonlinear pattern, we examined the distribution of patients and 28-day all-cause mortality events across SHR levels. Across SHR quartiles, 28-day all-cause mortality rates were 7.5%, 7.2%, 9.3%, and 25.8% from Q1 to Q4, respectively. The highest SHR decile (SHR 1.45–4.42) included 249 patients and 103 deaths, with a mortality rate of 41.4%; in contrast, the lowest SHR decile (SHR 0.19–0.67) included 265 patients and 18 deaths, with a mortality rate of 6.8%. At the very low SHR extreme (SHR ≤0.41), only 28 patients and 3 deaths were observed, indicating that estimates in this range should be interpreted cautiously. After excluding patients with SHR values below the 1st and above the 99th percentiles, the association between the highest SHR quartile and 28-day all-cause mortality remained significant (adjusted HR 2.130, 95% CI 1.467-3.094, P < 0.001). Similar findings were observed after excluding SHR values below the 2.5th and above the 97.5th percentiles (HR 1.857, 95% CI 1.269-2.717, P = 0.001) and below the 5th and above the 95th percentiles (HR 1.805, 95% CI 1.186-2.746, P = 0.006). These results are provided in **Supplementary Table 5**-**S7**.

### Exploratory Boruta feature-importance analysis

**Figure 4:**
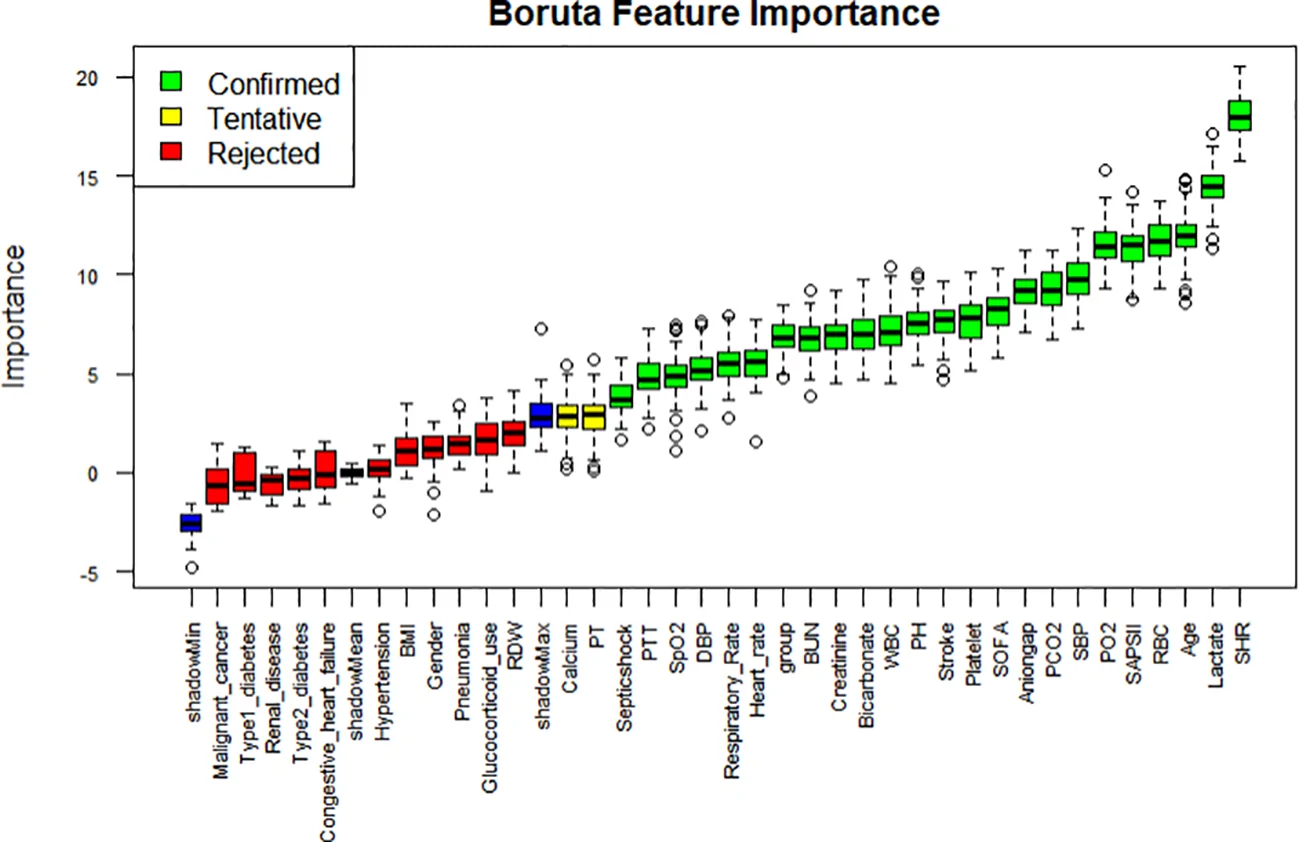
Feature selection based on the Boruta algorithm.

**Figure 4** shows the exploratory Boruta feature-importance results. Variables in the green area were classified as important, variables in the red area were classified as unimportant, and variables in the yellow area were classified as tentative. SHR ranked among the important predictors of 28-day all-cause mortality in patients with AHRF, but these feature-importance results should be interpreted as predictive and exploratory rather than causal.

### Subgroup analysis

**Figure 5:**
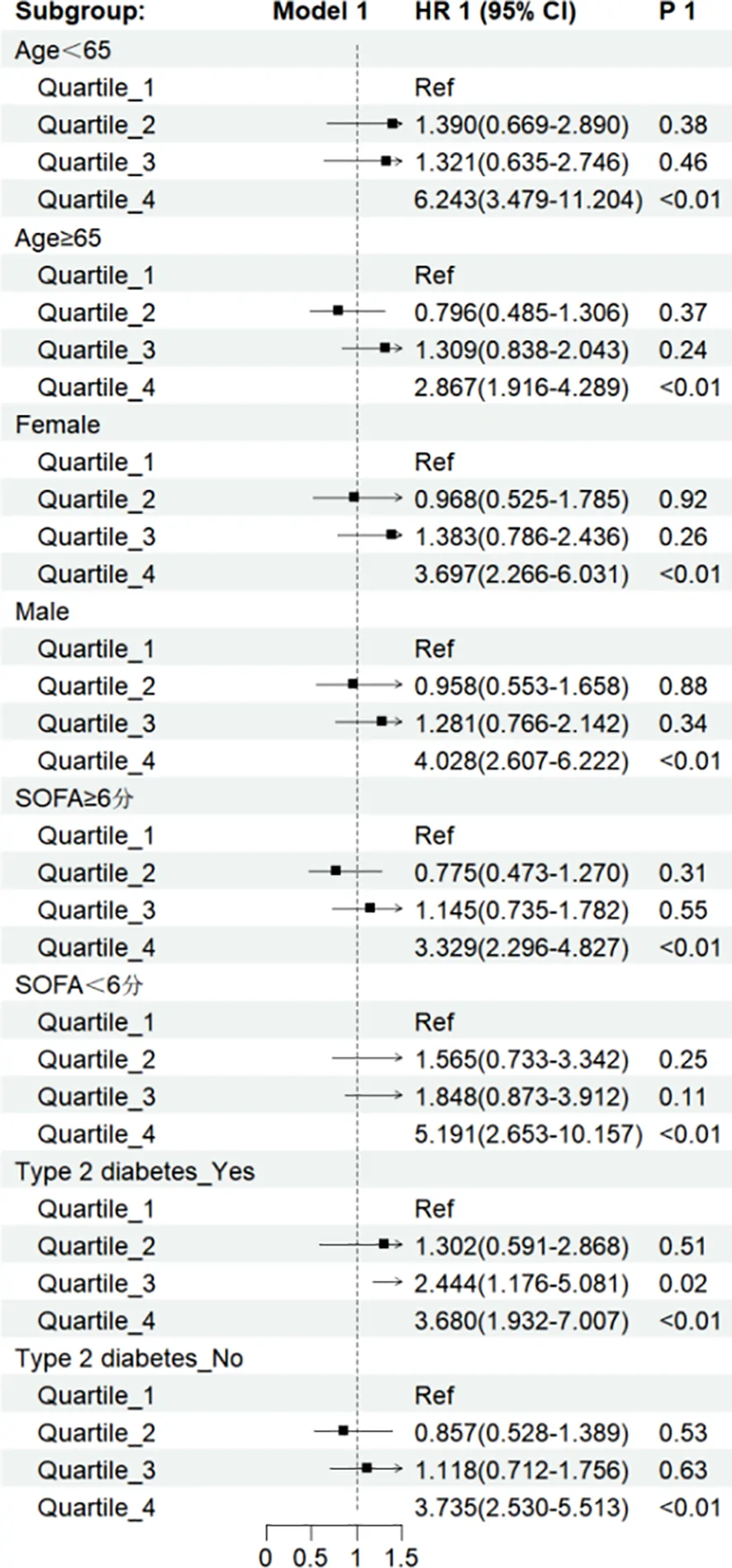
Subgroup analysis for 28-day all-cause mortality. Quartile 1 (0.19 ≤ SHR < 0.84), Quartile 2 (0.84 ≤ SHR < 1.02), Quartile 3 (1.02 ≤ SHR < 1.20), and Quartile 4 (1.20 ≤ SHR ≤ 4.42).

Subgroup analyses were performed to evaluate the association between SHR quartiles and 28-day all-cause mortality across clinically relevant strata, including age, sex, SOFA score, and type 2 diabetes status (**Figure 5**). The highest SHR quartile was generally associated with increased 28-day all-cause mortality across these strata. However, these subgroup analyses were exploratory and should not be interpreted as definitive evidence that baseline characteristics modify, or do not modify, the SHR-mortality association.

### Establishment and validation of machine learning models

**Figure 6:**
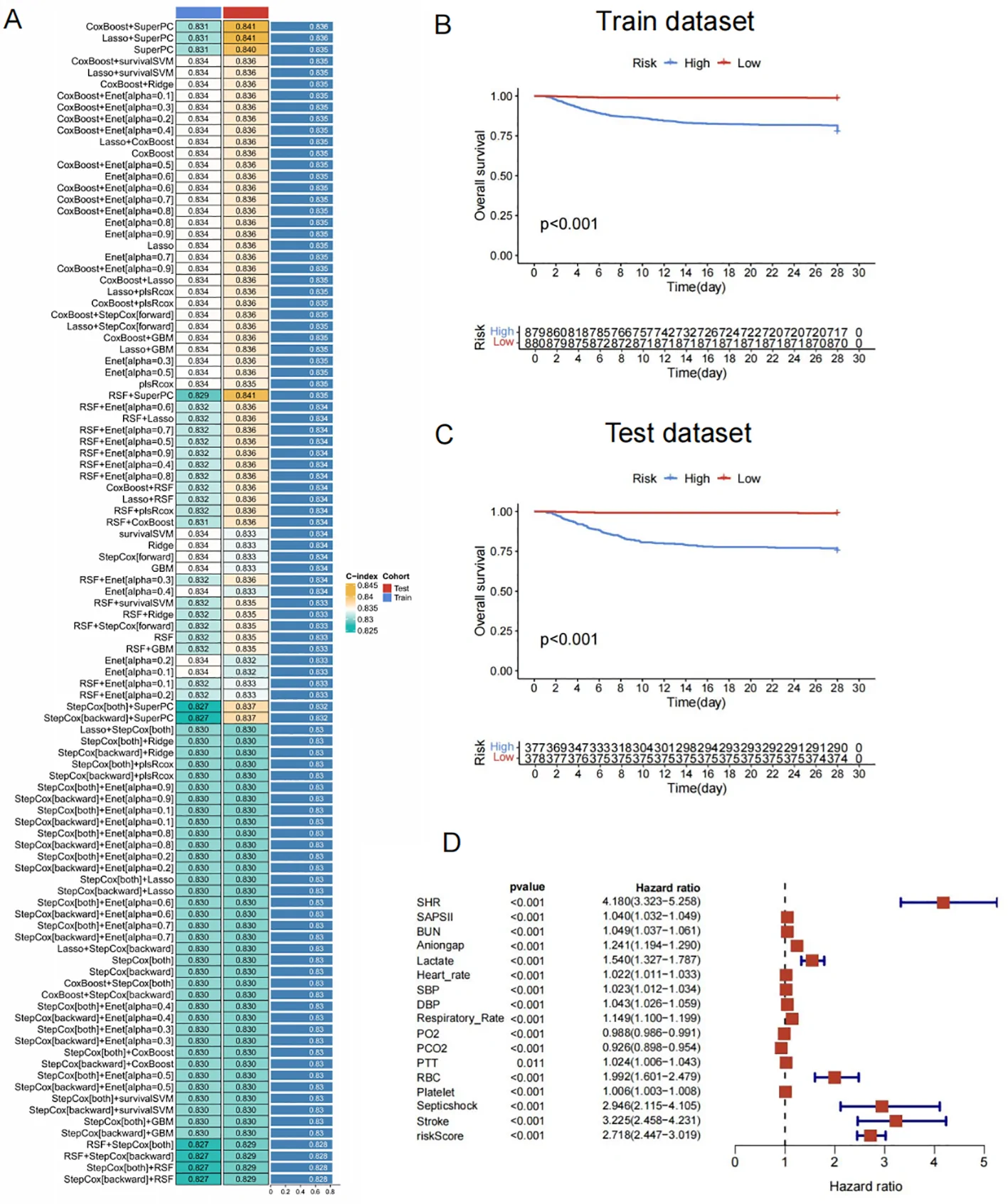
Predictive modeling and risk stratification. **(A)** Performance comparison of candidate predictive models and model combinations in the training and validation cohorts. **(B)** Kaplan–Meier survival curves for high- and low-risk groups in the training cohort. **(C)** Kaplan–Meier survival curves for high- and low-risk groups in the validation cohort. **(D)** Forest plot showing the associations between risk score and prognosis-related clinical features.

In the secondary predictive analysis, SHR demonstrated better discrimination than SOFA for predicting 28-day all-cause mortality. The AUC was 0.690 for SHR and 0.602 for SOFA, with an AUC difference of 0.088 by the DeLong test (P < 0.001). Adding SHR to SOFA improved discrimination, increasing the AUC to 0.706 (AUC difference versus SOFA = 0.105, P < 0.001), and the Brier score decreased from 0.106 for SOFA alone to 0.099 for SOFA + SHR. Compared with SHR alone, SOFA + SHR showed a modest, non-significant increase in AUC (AUC difference = 0.017, P = 0.101). In the machine-learning analysis, the final selected model was CoxBoost + SuperPC. The model retained 18 features, with an optimized CoxBoost penalty of 1000, an optimal boosting step of 81, and a SuperPC threshold of 1.524. The apparent C-index in the training cohort was 0.814. After bootstrap optimism correction, the optimism-corrected C-index was 0.803. In the independent validation cohort, the locked model achieved a C-index of 0.826. CoxBoost + SuperPC achieved a 28-day time-dependent AUC of 0.845 in the validation cohort and an AUC of 0.847 in the supplementary binary-endpoint DeLong analysis. The predictive-model comparison and risk-stratification results are shown in **Figure 6**. These results are summarized in **Supplementary Table 11**-**S15**, **S17**, and **S18**. 

### Consensus clustering analysis

Consensus clustering identified three major sample clusters. The cumulative distribution function (CDF) and delta area plots showed changes in clustering stability across k = 2 to 6 (**Supplementary Figure 2**). The consensus matrix heatmap showed clearer boundaries at k = 3 (**Supplementary Figure 3**). The PAC value was lowest at k = 3 (PAC = 0.057; **Supplementary Table 3**), supporting the selection of three clusters as the exploratory phenotyping solution.

Consensus clustering identified three exploratory clinical phenotypes in patients with AHRF, and t-SNE plots were used to visualize phenotype separation (**Supplementary Figure 5**). The characteristics of the three phenotypes are presented in **Table 4**. Phenotype I showed relatively lower severity and more stable metabolic and inflammatory profiles. Phenotype II showed intermediate clinical severity with moderate metabolic and inflammatory abnormalities. Phenotype III showed a higher-severity clinical profile with more pronounced organ dysfunction, hypoxemia, and inflammatory abnormalities. These clusters should be interpreted as descriptive clinical phenotypes rather than validated biological subtypes.

**Table 4:** Characteristics of the three phenotypes.

Characteristic	N	Overall N = 2,514	Phenotype-I N = 1,508	Phenotype-II N = 523	Phenotype-III N = 483	*P* value
Age	2,514	67.2±13.1	68.8±11.7	63.0±14.4	66.7±14.7	<0.001
SHR	2,514	1.0±0.3	1.0±0.3	1.1±0.3	1.3±0.5	<0.001
Gender	2,514					0.013
Male		1,655(66%)	1,016(67%)	316(60%)	323(67%)	
Female		859(34%)	492(33%)	207(40%)	160(33%)	
BMI	2,514	29.3±7.7	29.4±6.7	29.3±9.3	28.8±8.6	0.300
SOFA	2,514	6.9±2.8	6.6±2.3	5.4±2.3	9.6±3.0	<0.001
SAPSII	2,514	39.2±12.8	36.7±10.6	35.4±10.9	51.4±13.7	<0.001
BUN	2,514	19.2±9.3	16.4±6.4	18.0±8.9	29.3±10.3	<0.001
Creatinine	2,514	1.1±0.5	1.0±0.3	1.0±0.4	1.6±0.6	<0.001
Aniongap	2,514	12.9±3.0	11.7±2.4	13.4±2.5	16.0±2.9	<0.001
Bicarbonate	2,514	22.9±3.0	23.3±2.3	23.4±3.2	21.0±3.6	<0.001
Lactate	2,514	2.0±0.8	2.0±0.7	1.6±0.6	2.6±1.1	<0.001
Calcium	2,514	1.1±0.1	1.2±0.0	1.1±0.1	1.1±0.1	<0.001
Heart rate	2,514	83.6±12.0	81.1±9.9	85.3±13.4	89.4±14.2	<0.001
SBP	2,514	113.8±11.8	111.3±7.9	126.8±12.4	107.4±10.9	<0.001
DBP	2,514	59.3±8.2	57.1±6.4	67.0±8.2	57.9±8.2	<0.001
Respiratory Rate	2,514	19.1±2.9	18.3±2.4	19.9±3.0	20.7±3.2	<0.001
pH	2,514	7.4±0.0	7.4±0.0	7.4±0.1	7.4±0.1	<0.001
PaO2	2,514	184.1±62.1	212.9±53.0	138.1±48.3	143.6±47.5	<0.001
PaCO2	2,514	40.1±4.6	40.5±3.7	39.9±5.9	39.2±5.4	<0.001
SpO2	2,514	96.5±1.2	96.6±1.0	96.6±1.5	96.3±1.5	<0.001
PTT	2,514	32.9±6.8	32.7±5.8	30.4±6.4	36.3±8.4	<0.001
PT	2,514	14.1±1.7	14.3±1.5	13.3±1.7	14.6±2.1	<0.001
WBC	2,514	12.9±4.4	12.7±4.0	12.6±4.4	13.9±5.3	<0.001
RBC	2,514	3.6±0.6	3.4±0.5	4.0±0.6	3.5±0.7	<0.001
RDW	2,514	14.1±1.2	13.9±1.1	14.2±1.2	14.7±1.2	<0.001
Platelet	2,514	169.8±58.2	152.8±47.2	212.7±55.4	176.5±66.4	<0.001
Glucocorticoid use	2,514					<0.001
No		2,427(97%)	1,481(98%)	489(93%)	457(95%)	
Yes		87(3.5%)	27(1.8%)	34(6.5%)	26(5.4%)	
Hypertension	2,514					<0.001
No		1,500(60%)	867(58%)	299(57%)	334(69%)	
Yes		1,014(40%)	641(42%)	224(43%)	149(31%)	
Pneumonia	2,514					<0.001
No		1,880(75%)	1,297(86%)	310(59%)	273(57%)	
Yes		634(25%)	211(14%)	213(41%)	210(43%)	
Septic shock	2,514					<0.001
No		2,298(91%)	1,483(98%)	500(96%)	315(65%)	
Yes		216(8.6%)	25(1.7%)	23(4.4%)	168(35%)	
Stroke	2,514					<0.001
No		2,030(81%)	1,390(92%)	220(42%)	420(87%)	
Yes		484(19%)	118(7.8%)	303(58%)	63(13%)	
Type 1 diabetes	2,514					0.7
No		2,489(99%)	1,494(99%)	516(99%)	479(99%)	
Yes		25(1.0%)	14(0.9%)	7(1.3%)	4(0.8%)	
Type 2 diabetes	2,514					0.004
No		1,973(78%)	1,206(80%)	415(79%)	352(73%)	
Yes		541(22%)	302(20%)	108(21%)	131(27%)	
Renal disease	2,514					<0.001
No		1,929(77%)	1,208(80%)	425(81%)	296(61%)	
Yes		585(23%)	300(20%)	98(19%)	187(39%)	
Malignant cancer	2,514					0.2
No		2,314(92%)	1,396(93%)	471(90%)	447(93%)	
Yes		200(8.0%)	112(7.4%)	52(9.9%)	36(7.5%)	
Congestive heart failure	2,514					<0.001
No		1,452(58%)	949(63%)	330(63%)	173(36%)	
Yes		1,062(42%)	559(37%)	193(37%)	310(64%)	
28-day all-cause mortality	2,514					<0.001
No		2,200(88%)	1,467(97%)	395(76%)	338(70%)	
Yes		314(12%)	41(2.7%)	128(24%)	145(30%)	
28-day survival time (days)	2,514	25.8±6.7	27.6±3.0	23.8±8.8	22.4±9.7	<0.001

### Relationships between phenotypes and outcomes

**Figure 7:**
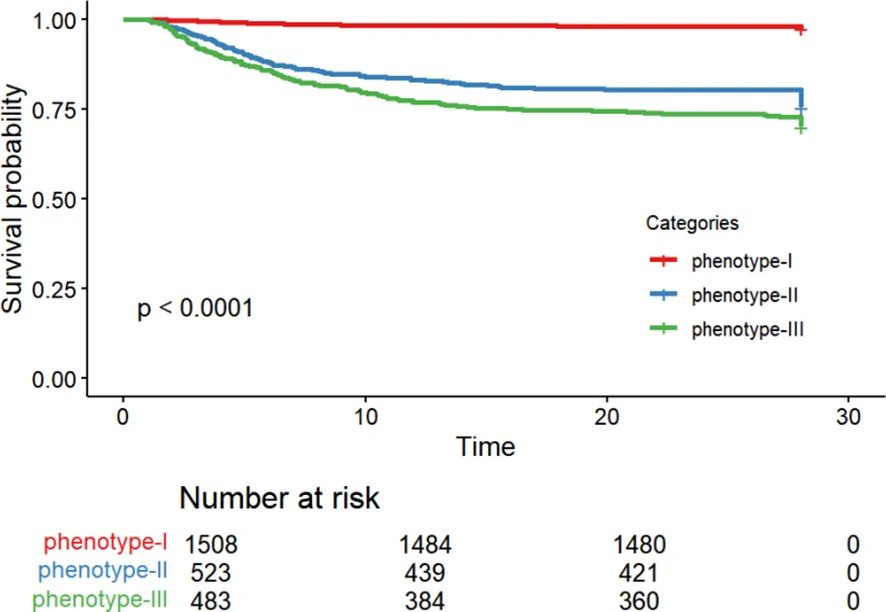
28-day KM survival curve based on different phenotypes.

Mortality outcomes differed across the three phenotypes. Phenotype III had the highest 28-day all-cause mortality rate (30.0%), followed by Phenotype II (24.5%) and Phenotype I (2.7%) (P < 0.001). Phenotype III also had the shortest mean 28-day survival time (22.4 ± 9.7 days), compared with Phenotype I (27.6 ± 3.0 days) and Phenotype II (23.8 ± 8.8 days) (P < 0.001; **Table 4**). Kaplan-Meier survival analysis showed significant survival differences among the phenotypes (**Figure 7**). These findings suggest that the clusters captured clinically meaningful differences in illness severity and prognosis, but they should not be interpreted as validated biological subtypes.

### Heterogeneity of SHR effects in different phenotypes

**Figure 8:**
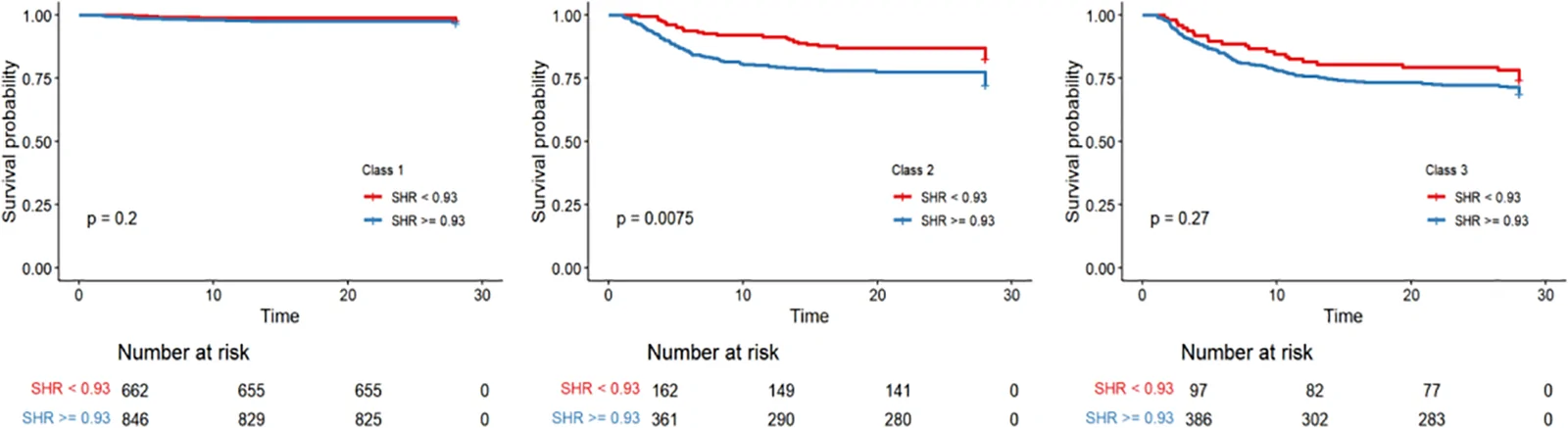
Kaplan–Meier survival curves for 28-day survival stratified by SHR group (<0.93 versus ≥0.93) within Class 1, Class 2, and Class 3.

The association between SHR and mortality was further explored across the three phenotypes. Each phenotype was divided into high- and low-SHR groups using the data-derived threshold of 0.93. In the primary adjusted model, SHR ≥0.93 was associated with 28-day all-cause mortality in Phenotype II (HR = 1.696, 95% CI: 1.104-2.605, P = 0.016), but not in Phenotype I (HR = 1.342, 95% CI: 0.675-2.667, P = 0.402) or Phenotype III (HR = 1.146, 95% CI: 0.730-1.798, P = 0.554; **Table 5** and **Figure 8**). However, formal interaction testing showed that the dichotomized SHR × phenotype interaction was not statistically significant (P for interaction = 0.456), whereas the continuous SHR × phenotype interaction suggested possible heterogeneity (P for interaction = 0.031). Therefore, phenotype-specific analyses should be interpreted as exploratory and hypothesis-generating. , 

**Table 5:** Cox regression model for 28-day all-cause mortality.

Categories	HR	Lower bound 95% CI	Upper bound 95% CI	P value
Phenotype I				
SHR < 0.93	Reference	Reference	Reference	Reference
SHR ≥ 0.93	1.342	0.675	2.667	0.402
Phenotype II				
SHR < 0.93	Reference	Reference	Reference	Reference
SHR ≥ 0.93	1.696	1.104	2.605	0.016
Phenotype III				
SHR < 0.93	Reference	Reference	Reference	Reference
SHR ≥ 0.93	1.146	0.730	1.798	0.554

## Discussion

To our knowledge, this study is among the first to investigate the association between the stress hyperglycemia ratio (SHR) and adverse outcomes in patients with acute hypoxemic respiratory failure (AHRF). Our findings showed that elevated SHR was associated with increased 28-day all-cause mortality in this population. This association remained generally consistent across several statistical models and sensitivity analyses, suggesting that SHR may serve as a potential prognostic marker for risk stratification in patients with AHRF. However, given the retrospective observational design of this study, these findings should be interpreted as statistical associations rather than evidence of causality.

Hyperglycemia has been reported to be associated with adverse outcomes in patients with respiratory failure and other critical illnesses. Previous studies have suggested that hyperglycemia may be related to impaired immune response, systemic inflammation, oxidative stress, and endothelial dysfunction, which could contribute to worse outcomes in critically ill patients (9, 10). In patients with acute respiratory failure, acute metabolic stress and hyperglycemia may also be associated with the formation of advanced glycation end products and other metabolic abnormalities (11). However, these biological pathways were not directly measured in the present study. Therefore, our findings do not establish that SHR causes immune dysfunction, oxidative stress, endothelial injury, or lung injury. Instead, SHR should be interpreted as a clinically accessible marker that may reflect the severity of acute metabolic stress in patients with AHRF.

It is also important to recognize that admission glucose alone may not adequately distinguish acute stress hyperglycemia from chronic dysglycemia. SHR incorporates HbA1c and therefore reflects acute glucose elevation relative to a patient’s estimated chronic glycemic background (5, 7). Previous studies have reported that elevated SHR is associated with adverse outcomes in diabetes/prediabetes, sepsis, and acute coronary syndrome populations (12–14). Our findings extend these observations to patients with AHRF, suggesting that SHR may provide prognostic information in this critically ill population. Nevertheless, SHR remains a derived biomarker, and its biological meaning should be interpreted cautiously.

The restricted cubic spline analysis suggested a possible nonlinear association between SHR and 28-day all-cause mortality. However, the exact shape of this relationship should not be overinterpreted. Although the evidence for increased mortality risk at higher SHR levels was consistent across categorical Cox regression and sensitivity analyses, the lower SHR range contained fewer events and wider uncertainty. Therefore, our results support a nonlinear association between SHR and mortality, with the most stable evidence indicating increased risk among patients with elevated SHR. Further studies are needed to validate the possible nonlinear pattern, particularly in the lower SHR range.

The machine-learning analysis was intended to complement, rather than replace, the conventional regression analyses. The regression models addressed the association between SHR and 28-day all-cause mortality, whereas the machine-learning analysis evaluated whether SHR and routinely available clinical variables could contribute to risk prediction. The comparison with SOFA suggested that SHR captured prognostic information not fully reflected by conventional illness severity scoring. Although the CoxBoost + SuperPC model showed acceptable performance after optimism correction and in the independent validation cohort, these findings should be interpreted as exploratory predictive results and require prospective external validation before clinical implementation.

Several biologically plausible explanations may underlie the observed association between elevated SHR and mortality. Prior studies have shown that acute hyperglycemia may affect innate immune responses, including neutrophil migration, phagocytosis, and microbial killing, thereby increasing susceptibility to infection (15). Hyperglycemia has also been linked to increased release of pro-inflammatory cytokines, such as interleukin-6 and tumor necrosis factor-alpha, and to oxidative stress and endothelial dysfunction (15, 16). These mechanisms may be relevant in patients with AHRF, in whom inflammation, hypoxemia, and organ dysfunction are common. However, inflammatory cytokines, oxidative stress markers, endothelial function, and endocrine stress-response markers were not directly measured in our study. Therefore, these mechanisms should be regarded as biologically plausible hypotheses based on previous literature rather than conclusions directly demonstrated by our data.

Population-level metabolic risk factors may influence the interpretation and generalizability of SHR. Previous population-based studies have shown that cardiometabolic risk profiles, including low high-density lipoprotein cholesterol, may differ across ethnic and demographic groups (17). Therefore, the prognostic significance of SHR should not be assumed to be identical across all ethnic, regional, and healthcare-system settings. Because the present study was based on a single-center ICU database, regional susceptibility and geographic heterogeneity could not be directly assessed. Future multicenter and multi-region epidemiological cohorts are needed to evaluate the transportability, calibration, and generalizability of SHR-based risk prediction models across diverse populations. In addition, large language models may facilitate extraction of clinically relevant information from unstructured electronic health records and support phenotype differentiation in future risk-stratification workflows. However, rigorous validation, privacy protection, and bias assessment remain necessary before clinical implementation.

Although diabetic peripheral neuropathy was not evaluated in the present AHRF cohort, chronic glycemic complications may contribute to the broader interpretation of metabolic risk. SHR in this study was assessed as a marker of acute metabolic stress and mortality risk rather than as a biomarker intended to guide the management of diabetic peripheral neuropathy. Diabetic peripheral neuropathy may be evaluated in future studies as a clinically relevant manifestation of chronic glycemic injury and microvascular dysfunction (18). Additional glycemic and metabolic risk factors, including HbA1c, admission glucose, glycemic variability, hypoglycemia, diabetes-related complications, and inflammatory biomarkers, may also provide prognostic information beyond SHR and warrant further investigation.

From a clinical perspective, SHR should be interpreted as a risk-stratification marker rather than a treatment-directing biomarker. Elevated SHR may help identify AHRF patients who require closer monitoring and reassessment of acute metabolic stress, illness severity, infection or shock status, and glycemic fluctuations. However, because this was an observational study, our findings demonstrate prognostic association rather than therapeutic effect. Therefore, SHR should not independently determine glycemic targets, insulin treatment strategies, or other treatment decisions, and the present study does not support an SHR-guided glucose-lowering protocol. Future prospective studies, external validation, and clinical impact studies are needed to determine whether SHR-guided risk stratification can improve treatment decision-making.

Consensus clustering identified three clinical phenotypes with distinct clinical profiles and mortality risks. Phenotype I was characterized by relatively lower severity and more stable metabolic and inflammatory profiles. Phenotype II showed intermediate clinical severity with moderate metabolic and inflammatory abnormalities. Phenotype III represented a higher-severity clinical phenotype with more pronounced organ dysfunction, hypoxemia, and inflammatory abnormalities. Consensus clustering was performed as an exploratory, data-driven clinical phenotyping analysis. The resulting clusters should be interpreted as descriptive clinical phenotypes rather than validated biological subtypes. Because several variables used for clustering also reflect illness severity, the differences in prognosis across phenotypes should be interpreted cautiously.

The phenotype-specific analysis suggested that the prognostic value of SHR may vary across clinical phenotypes. In Phenotype II, SHR showed clearer mortality stratification, whereas this association was not statistically significant in Phenotype I or Phenotype III when SHR was dichotomized at 0.93. This finding should be interpreted cautiously. One possible explanation is that Phenotype II represents an intermediate-risk subgroup in which stress hyperglycemia may provide additional prognostic information beyond overall disease severity. In contrast, patients in Phenotype III had more severe hypoxemia, inflammation, and organ dysfunction, and their mortality risk may have been primarily driven by overall illness severity. Under such circumstances, the incremental prognostic contribution of SHR may be statistically attenuated. This interpretation remains exploratory and should not be regarded as evidence of a causal mechanism.

Importantly, formal interaction testing suggested that phenotype-related heterogeneity depended on how SHR was modeled. When SHR was dichotomized at 0.93, the interaction between SHR category and phenotype was not statistically significant. When SHR was modeled as a continuous variable, the SHR × phenotype interaction was statistically significant. These findings suggest possible phenotype-related heterogeneity in the SHR–mortality association, but they also indicate that dichotomizing SHR may oversimplify its relationship with outcome. Therefore, the phenotype-specific findings should be considered hypothesis-generating and require validation in independent cohorts.

This study has several limitations. First, as a retrospective analysis based on the MIMIC-IV database, causal inference cannot be established, and residual confounding may remain despite multivariable adjustment. Second, because SHR calculation requires both glucose and HbA1c measurements, selection bias may have been introduced by excluding patients without these laboratory data, including potentially very severe patients who died before blood sampling could be completed. Third, the phenotypes identified by consensus clustering were derived from clinical and laboratory variables within a single retrospective database. Because some clustering variables also reflect disease severity and prognosis, the phenotype-outcome associations may be partly tautological. These phenotypes should therefore be interpreted as exploratory clinical patterns and require external validation before being considered stable biological subtypes. Fourth, although machine-learning models and clustering analyses provided additional prognostic information and the final locked model was evaluated in an independent validation cohort, the validation cohort was derived from the same database. External prospective validation is still required before clinical application. Fifth, because this study was based on a single-center ICU database, regional heterogeneity and geographic transportability of SHR-based risk prediction could not be directly assessed, highlighting the need for future multicenter epidemiological validation. Finally, laboratory assay platforms, reagent lots, and potential batch effects could not be fully assessed in this retrospective database study.

## Conclusion

In conclusion, SHR was associated with 28-day all-cause mortality in patients with AHRF and may serve as a potential prognostic marker for risk stratification. The most consistent evidence supported an increased mortality risk among patients with elevated SHR. Exploratory phenotype-specific analyses suggested that the prognostic utility of SHR may vary across clinical phenotypes, but these findings should be interpreted cautiously and require external validation. Future prospective studies are needed to confirm the prognostic value of SHR and to determine whether SHR is directly involved in disease progression or primarily reflects underlying illness severity.

## Data Availability

The data analyzed in this study are available from the MIMIC-IV database to credentialed researchers who complete the required training and sign the applicable data-use agreement. Because of these data-use restrictions, the raw data cannot be publicly shared by the authors. Further inquiries may be directed to the corresponding authors.
