# Supplementary material for: Nonlinear association between stress hyperglycemia ratio and mortality in AHRF: validation through consensus clustering and supervised machine learning models
Source: Front Endocrinol (Lausanne). 2026 Sep 1;17:1810713. doi: 10.3389/fendo.2026.1810713 (PMC13574610; doi:10.3389/fendo.2026.1810713)
Supplement: Supplementary file 1 [file DataSheet1.docx]

**Nonlinear Association Between Stress Hyperglycemia Ratio and Mortality in AHRF: Validation Through Consensus Clustering and Supervised Machine Learning Models**

Qi Zhang1,2†; Tao Wang3†; Haoyue Wang4†; Liye Ji1,2; Yang Zhao5; Xiaoyong Geng6; Zhiyong Wang1,2; Yaoyao Tang1,2*, Mingxing Fang1,2*

^1^Department of Critical Care Medicine, Hebei Medical University Third hospital, Hebei, China.

^2^Critical Disease Data Analysis and Intelligent Diagnosis and Treatment Engineering Research Center of Hebei Province, Hebei Medical University Third hospital, Hebei, China.

^3^Department of medical imaging, The People’s hospital of Guizhou, Yunnan, China.

4Applied Statistics, College of Science, Hebei University of Technology, Tianjin, China.

5Department of endocrinology, Shijiazhuang Traditional Chinese Hospital, Hebei, China.

6Department of Cardiology, The Third Hospital of Hebei Medical University, Shijiazhuang, Hebei, China.

* Corresponding author: m18533112886@hebmu.edu.cn (FMX)

^†^These authors contributed equally to this work.

**Additional file**

Supplementary Methods. Additional methodological details for the revised analyses.

Supplementary Results. Additional textual results for the revised analyses.

**Figure S1**. The percentage of missing data for various clinical variables in the study cohort.

**Figure S2**. (A) Consensus cumulative distribution function (CDF) plots for cluster numbers (k) ranging from 2 to 6. (B) Delta area curves of the consensus CDF, illustrating the relative change in clustering stability for k = 2 to 6.

**Figure S3**. The consensus matrix heatmap from consensus clustering analysis displays the agreement values for each cluster (k) in patients with AHRF, represented on a white-to-blue color scale.

**Figure S4**. The bar plot of consensus cluster analysis.

**Figure S5**. T-SNE visualization of phenotype assignments by consensus clustering.

Table S1. The variance inflation factors of variables.

Table S2. The features included in CoxBoost + SuperPC model.

Table S3. PAC values using the predetermined boundary of (0.1, 0.9) as the definition for ambiguously clustered pairs.

Table S4. Missingness summary for candidate variables.

Table S5. Distribution of 28-day all-cause mortality events across SHR quartiles.

Table S6. Distribution of 28-day all-cause mortality events at SHR extremes.

Table S7. Sensitivity analyses excluding extreme SHR values.

Table S8. Association between SHR quartiles and 28-day all-cause mortality across Cox models.

Table S9. Sensitivity analyses using alternative outlier-handling strategies.

Table S10. Sensitivity analyses comparing MissForest and MICE imputation.

Table S11. Predictive performance of SOFA, SHR, and SOFA + SHR for 28-day all-cause mortality.

Table S12. DeLong comparison of SOFA, SHR, and SOFA + SHR.

Table S13. Candidate machine-learning model grid.

Table S14. Pairwise comparison of 28-day time-dependent AUCs between CoxBoost + SuperPC and top-performing candidate models.

Table S15. Pairwise DeLong tests between CoxBoost + SuperPC and top-performing candidate models for the binary 28-day endpoint.

Table S16. Formal interaction testing between SHR and phenotype.

Table S17. Internal validation of the final CoxBoost + SuperPC model using bootstrap optimism correction and an independent validation cohort.

Table S18. Hyperparameter settings and final optimal values of the CoxBoost + SuperPC model.

**Supplementary Methods**

Additional analyses added in the supplementary methods to clarify cohort construction, preprocessing, covariate adjustment, sensitivity analyses, and the reproducibility of the predictive modeling workflow.

**Missing-data handling and imputation**

Missingness was evaluated after construction of the patient-level analytic dataset. Variables with >20% missingness were excluded before imputation to reduce imputation-related uncertainty. Variables with ≤20% missingness were retained and imputed using the MissForest algorithm. Outcome variables, including 28-day all-cause mortality and survival time, were not included in the imputation model to avoid outcome leakage. As a sensitivity analysis, the primary Cox regression analyses were repeated using multiple imputation by chained equations (MICE). A variable-level missingness summary is provided in Table S4, and the imputation sensitivity results are provided in Table S10.

**Outlier handling and sensitivity analyses**

Because extreme physiological and laboratory values in critically ill patients may represent true disease severity rather than erroneous measurements, the revised analysis did not rely solely on the 1.5 x IQR rule for excluding observations. Two alternative outlier-handling strategies were evaluated. First, only clinically implausible or physiologically impossible values were removed. Second, winsorization was applied to limit the influence of extreme values while retaining patients with clinically meaningful extreme measurements. The association between SHR quartiles and 28-day all-cause mortality was re-evaluated under both strategies using the same modeling framework. These results are summarized in Table S9.

**Covariate adjustment strategy**

A stepwise Cox modeling framework was used to reduce the risk of overadjustment. Model 1 was unadjusted. Model 2 was defined as the primary parsimonious model and adjusted for age, gender, BMI, SOFA score, type 2 diabetes, hypertension, pneumonia, septic shock, stroke, renal disease, malignant cancer, congestive heart failure, and glucocorticoid use. Acute physiological and laboratory variables, including lactate, bicarbonate, anion gap, blood gas parameters, coagulation markers, and hematologic markers, were not included in the primary model because they may represent downstream manifestations of acute illness or overlap with global disease severity. Model 3 was retained as an extended sensitivity model by adding broader physiological and laboratory variables to Model 2. Multicollinearity was assessed using variance inflation factors. The Cox model results are shown in Table S8.

**Assessment of SHR event distribution and extreme-value influence**

To evaluate the stability of the spline-based nonlinear pattern, the distribution of patients and 28-day all-cause mortality events was examined across SHR quartiles and at SHR extremes. Additional sensitivity analyses were performed by excluding patients with SHR values below the 1st and above the 99th percentiles, below the 2.5th and above the 97.5th percentiles, and below the 5th and above the 95th percentiles. These analyses were used to determine whether the association between elevated SHR and mortality was driven primarily by sparse values at the tails of the SHR distribution. The corresponding results are provided in Tables S5–S7.

**Incremental predictive value of SHR beyond SOFA**

Machine learning analysis was treated as a secondary predictive analysis. To directly assess whether SHR provided incremental predictive value beyond established severity scoring, three logistic regression models were compared for the binary 28-day all-cause mortality endpoint: SOFA alone, SHR alone, and SOFA + SHR. Model performance was assessed using the C-index/AUC, 95% confidence intervals for AUC, calibration intercept, calibration slope, and Brier score. AUCs were compared using DeLong tests. These results are shown in Tables S11 and S12.

**Machine learning model development, validation, and comparison**

A total of 101 candidate machine learning models or two-step model combinations were evaluated. The complete candidate model grid is listed in Table S13. For the final CoxBoost + SuperPC model, CoxBoost was used for feature selection and SuperPC was used for final survival prediction. The CoxBoost penalty parameter was optimized using optimCoxBoostPenalty with start.penalty = 500, and the optimal number of boosting steps was selected by 10-fold cross-validation using cv.CoxBoost with maxstepno = 500, K = 10, and type = "verweij". SuperPC was trained with type = "survival" and s0.perc = 0.5, and the threshold was selected by 10-fold cross-validation with n.threshold = 20, n.fold = 10, n.components = 3, and min.features = 5. The final selected values are summarized in Table S18.

Model performance was assessed using the C-index, 28-day area under the curve (AUC), calibration metrics, and Brier score. Bootstrap optimism correction was performed in the training cohort by repeating the complete modeling procedure, including CoxBoost-based feature selection and SuperPC model construction. The C-index was calculated in each bootstrap sample and in the original training cohort, and the mean optimism was subtracted from the apparent C-index to obtain the optimism-corrected C-index. The final locked model was then evaluated in an independent validation cohort that was not used for feature selection, model training, hyperparameter tuning, or cutoff determination. Pairwise comparisons between CoxBoost + SuperPC and top-performing candidate models were performed using 28-day time-dependent AUC comparison, with DeLong tests added as supplementary analyses for the binary 28-day endpoint. These analyses are reported in Tables S14, S15, and S17.

**Phenotype-specific analysis and formal interaction testing**

Consensus clustering was interpreted as an exploratory, data-driven clinical phenotyping analysis rather than a validated biological taxonomy. Phenotype-stratified Cox models using SHR ≥0.93 were retained as exploratory subgroup analyses. To formally assess possible phenotype-related heterogeneity, unified Cox proportional hazards models were fitted with interaction terms between phenotype and SHR. Interaction testing was performed for both dichotomized SHR and continuous SHR. The interaction results are shown in Table S16.

**Supplementary Results**

**Event distribution across SHR levels**

Across SHR quartiles, 28-day all-cause mortality rates were 7.5%, 7.2%, 9.3%, and 25.8% from Q1 to Q4, respectively. The highest SHR decile (SHR 1.45-4.42) included 249 patients and 103 deaths, with a mortality rate of 41.4%. In contrast, the lowest SHR decile (SHR 0.19-0.67) included 265 patients and 18 deaths, with a mortality rate of 6.8%. At the very low SHR extreme (SHR ≤0.41), only 28 patients and 3 deaths were observed, indicating that estimates in this range should be interpreted cautiously. The quartile and extreme-value event distributions are summarized in Tables S5 and S6.

**Sensitivity analyses excluding extreme SHR values**

After excluding patients with SHR values below the 1st and above the 99th percentiles, the association between the highest SHR quartile and 28-day all-cause mortality remained significant (adjusted HR 2.130, 95% CI 1.467-3.094, P < 0.001). Similar findings were observed after excluding SHR values below the 2.5th and above the 97.5th percentiles (HR 1.857, 95% CI 1.269-2.717, P = 0.001) and after excluding SHR values below the 5th and above the 95th percentiles (HR 1.805, 95% CI 1.186-2.746, P = 0.006). These results suggest that the association between elevated SHR and mortality was not driven solely by extreme SHR values. The results are shown in Table S7.

**Cox regression under the revised adjustment framework**

The association between SHR and 28-day all-cause mortality remained consistent across the revised Cox modeling framework. In Model 1, the unadjusted HR for Quartile 4 versus Quartile 1 was 3.892 (95% CI 2.806-5.398, P < 0.001). In Model 2, the primary parsimonious model, the HR was 2.972 (95% CI 2.108-4.189, P < 0.001). In Model 3, the extended sensitivity model, the HR was 2.102 (95% CI 1.464-3.018, P < 0.001). The maximum VIF was 1.22 in Model 2 and 3.11 in Model 3, suggesting no substantial statistical multicollinearity. Detailed results are shown in Table S8.

**Outlier-handling sensitivity analyses**

The association between elevated SHR and 28-day all-cause mortality remained robust under alternative outlier-handling strategies. After removing clinically implausible values, the HR for Quartile 4 versus Quartile 1 was 4.035 (95% CI 2.922-5.571, P < 0.001) in the unadjusted model and 2.859 (95% CI 1.975-4.138, P < 0.001) in the primary parsimonious model. After winsorization, the results were unchanged, with an HR of 4.035 (95% CI 2.922-5.571, P < 0.001) in the unadjusted model and 2.859 (95% CI 1.975-4.138, P < 0.001) in the primary parsimonious model. These results are summarized in Table S9.

**Missing-data and imputation sensitivity analyses**

The missingness summary showed that ALT, total bilirubin, AST, ALP, fibrinogen, hemoglobin, sodium, and potassium exceeded the 20% missingness threshold and were excluded before imputation. The remaining variables with ≤20% missingness were imputed using MissForest. When the primary Cox models were repeated using MICE, the association between elevated SHR and 28-day all-cause mortality remained consistent with the MissForest-based analysis. Missingness details and imputation sensitivity results are presented in Tables S4 and S10.

**Incremental predictive value of SHR**

SHR demonstrated better discrimination than SOFA for predicting 28-day all-cause mortality. The AUC was 0.690 for SHR and 0.602 for SOFA, with an AUC difference of 0.088 by DeLong test (P < 0.001). Adding SHR to SOFA improved model discrimination, increasing the AUC to 0.706 (AUC difference versus SOFA = 0.105, P < 0.001). The Brier score also decreased from 0.106 for SOFA alone to 0.099 for SOFA + SHR. Compared with SHR alone, SOFA + SHR showed a modest, non-significant increase in AUC (AUC difference = 0.017, P = 0.101). These results are shown in Tables S11 and S12.

**Machine learning model performance and validation**

The final selected machine learning model was CoxBoost + SuperPC. The model retained 18 features, with an optimized CoxBoost penalty of 1000, an optimal boosting step of 81, and a SuperPC threshold of 1.524. The apparent C-index in the training cohort was 0.814. After bootstrap optimism correction, the estimated mean optimism was 0.011, yielding an optimism-corrected C-index of 0.803. In the independent validation cohort, the locked model achieved a C-index of 0.826. These findings are summarized in Tables S17 and S18.

**Pairwise model comparisons**

CoxBoost + SuperPC achieved a 28-day time-dependent AUC of 0.845 in the validation cohort. Its performance was comparable to Lasso + SuperPC and SuperPC alone, but it significantly outperformed the survival-SVM-based candidate models, including CoxBoost + survivalSVM and Lasso + survivalSVM. Supplementary DeLong tests for the binary 28-day endpoint yielded similar findings, with CoxBoost + SuperPC achieving an AUC of 0.847. Detailed pairwise comparisons are provided in Tables S14 and S15.

**Phenotype-specific SHR analysis and interaction testing**

In phenotype-stratified analyses using SHR ≥0.93, elevated SHR was associated with 28-day all-cause mortality in Phenotype II but not in Phenotypes I or III. In the primary adjusted model, the HR for SHR ≥0.93 was 1.342 in Phenotype I (95% CI 0.675-2.667, P = 0.402), 1.696 in Phenotype II (95% CI 1.104-2.605, P = 0.016), and 1.146 in Phenotype III (95% CI 0.730-1.798, P = 0.554). However, the formal interaction test between dichotomized SHR and phenotype in a unified Cox model was not statistically significant (P for interaction = 0.456). The continuous SHR × phenotype interaction suggested possible heterogeneity (P for interaction = 0.031), but this finding should be interpreted cautiously given the nonlinear SHR-mortality relationship and the exploratory nature of phenotype-specific analyses. Interaction results are summarized in Table S16.

**Figure S1.** The percentage of missing data for various clinical variables in the study cohort.

**Figure S2.** (A) Consensus cumulative distribution function (CDF) plots for cluster numbers (k) ranging from 2 to 6. (B) Delta area curves of the consensus CDF, illustrating the relative change in clustering stability for k = 2 to 6.

**Figure S3.** The consensus matrix heatmap from consensus clustering analysis displays the agreement values for each cluster (k) in patients with AHRF, represented on a white-to-blue color scale.

**Figure S4.** The bar plot of consensus cluster analysis.

**Figure S5.** T-SNE visualization of phenotype assignments by consensus clustering.

**Table S1.** The variance inflation factors of variables.

| Variables | VIF | Variables | VIF |
| --- | --- | --- | --- |
| Age | 1.460 | SHR | 1.184 |
| Gender1 | 1.198 | BMI | 1.058 |
| SOFA | 2.103 | Heart rate | 1.311 |
| BUN | 2.505 | Creatinine | 3.030 |
| Aniongap | 1.709 | Bicarbonate | 2.917 |
| Lactate | 1.545 | Calcium | 1.220 |
| SBP | 1.969 | DBP | 1.792 |
| Respiratory Rate | 1.253 | PH | 2.811 |
| PO2 | 1.373 | PCO2 | 2.519 |
| SpO2 | 1.211 | PTT | 1.209 |
| PT | 1.367 | WBC | 1.173 |
| RBC | 1.494 | RDW | 1.262 |
| Platelet | 1.400 | Glucocorticoid use1 | 1.071 |
| Hypertension1 | 1.133 | Pneumonia1 | 1.184 |
| Septic shock1 | 1.256 | Stroke1 | 1.399 |
| Type1 diabetes1 | 1.054 | Type 2 diabetes1 | 1.168 |
| Renal disease1 | 1.678 | Malignant cancer1 | 1.057 |
| Congestive heart failure1 | 1.368 |  |  |

**Table S2** The features included in CoxBoost+SuperPC model.

| SHR | CoxBoost+SuperPC |
| --- | --- |
| SAPSII | CoxBoost+SuperPC |
| BUN | CoxBoost+SuperPC |
| Aniongap | CoxBoost+SuperPC |
| Lactate | CoxBoost+SuperPC |
| Heart rate | CoxBoost+SuperPC |
| SBP | CoxBoost+SuperPC |
| DBP | CoxBoost+SuperPC |
| Respiratory Rate | CoxBoost+SuperPC |
| PO2 | CoxBoost+SuperPC |
| PCO2 | CoxBoost+SuperPC |
| PTT | CoxBoost+SuperPC |
| RBC | CoxBoost+SuperPC |
| Platelet | CoxBoost+SuperPC |
| Septic shock | CoxBoost+SuperPC |
| Stroke | CoxBoost+SuperPC |

**Table S3** The PAC values using the predetermined boundary of (0.1, 0.9) as the definition for ambiguously clustered pairs.

| K=2 | K=3 | K=4 | K=5 | K=6 |
| --- | --- | --- | --- | --- |
| 0.364 | 0.057 | 0.378 | 0.341 | 0.263 |

**Table S4. Missingness summary for candidate variables.**

| **Column** | **Count** | **Missing Ratio (%)** |
| --- | --- | --- |
| ALT | 1573 | 62.57 |
| Bilirubin_Total | 1570 | 62.45 |
| AST | 1565 | 62.25 |
| ALP | 1545 | 61.46 |
| Fibrinogen | 948 | 37.71 |
| Hemoglobin | 765 | 30.43 |
| Sodium | 762 | 30.31 |
| Potassium | 592 | 23.55 |
| Calcium | 342 | 13.60 |
| Height | 290 | 11.54 |
| Lactate | 209 | 8.31 |
| Creatinine | 118 | 4.69 |
| PT | 112 | 4.46 |
| INR | 111 | 4.42 |
| RDW | 101 | 4.02 |
| BUN | 100 | 3.98 |
| PCO2 | 83 | 3.30 |
| PTT | 81 | 3.22 |
| PH | 68 | 2.70 |
| PO2 | 58 | 2.31 |
| Platelet | 58 | 2.31 |
| Bicarbonate | 38 | 1.51 |
| Aniongap | 17 | 0.68 |
| RBC | 11 | 0.44 |
| SOFA | 2 | 0.08 |
| lods | 2 | 0.08 |
| SAPSII | 2 | 0.08 |
| SIRS | 2 | 0.08 |
| Type 1 diabetes | 2 | 0.08 |
| SBP | 1 | 0.04 |
| DBP | 1 | 0.04 |
| HbAc1_avg | 0 | 0 |
| Glu_avg | 0 | 0 |
| Gender | 0 | 0 |
| Age | 0 | 0 |
| Weight | 0 | 0 |
| Heart_rate | 0 | 0 |
| MBP | 0 | 0 |
| Respiratory_Rate | 0 | 0 |
| SpO2 | 0 | 0 |
| WBC | 0 | 0 |
| Glucocorticoid use | 0 | 0 |
| Hypertension | 0 | 0 |
| Pneumonia | 0 | 0 |
| Septicshock | 0 | 0 |
| Stroke | 0 | 0 |
| Type2_diabetes | 0 | 0 |
| Renal_disease | 0 | 0 |
| Malignant_cancer | 0 | 0 |
| Congestive heart failure | 0 | 0 |

Variables with >20% missingness were excluded before imputation. Variables with ≤20% missingness were retained and imputed using MissForest, with MICE used as a sensitivity analysis.

**Table S5. Distribution of 28-day all-cause mortality events across SHR quartiles.**

| SHR group | N | 28-day deaths | Mortality rate |
| --- | --- | --- | --- |
| Q1: 0.19–0.83 | 612 | 46 | 7.5% |
| Q2: 0.84–1.01 | 642 | 46 | 7.2% |
| Q3: 1.02–1.19 | 624 | 58 | 9.3% |
| Q4: 1.20–4.42 | 636 | 164 | 25.8% |

**Table S6. Distribution of 28-day all-cause mortality events at SHR extremes.**

| SHR extreme | N | 28-day deaths | Mortality rate |
| --- | --- | --- | --- |
| ≤1st percentile, SHR ≤0.41 | 28 | 3 | 10.7% |
| ≤5th percentile, SHR ≤0.57 | 132 | 11 | 8.3% |
| ≥95th percentile, SHR ≥1.65 | 129 | 60 | 46.5% |
| ≥99th percentile, SHR ≥2.14 | 27 | 13 | 48.1% |

**Table S7. Sensitivity analyses excluding extreme SHR values.**

| **Sensitivity analysis** | **N / deaths** | **HR** | **95% CI** | **P value** |
| --- | --- | --- | --- | --- |
| Full cohort | 2514 / 314 | 2.109 | 1.469–3.029 | <0.001 |
| Excluding SHR <1st and >99th percentiles | 2464 / 299 | 2.130 | 1.467–3.094 | <0.001 |
| Excluding SHR <2.5th and >97.5th percentiles | 2395 / 279 | 1.857 | 1.269–2.717 | 0.001 |
| Excluding SHR <5th and >95th percentiles | 2267 / 245 | 1.805 | 1.186–2.746 | 0.006 |

The comparison is Q4 versus Q1.

**Table S8. Association between SHR quartiles and 28-day all-cause mortality across Cox proportional hazards models.**

| **Model** | **Categories** | **HR** | **95% CI** | **P value** |
| --- | --- | --- | --- | --- |
| Model 1 — unadjusted | Q1 | Reference | Reference | Reference |
| Model 1 — unadjusted | Q2 | 0.957 | 0.636–1.440 | 0.833 |
| Model 1 — unadjusted | Q3 | 1.257 | 0.853–1.851 | 0.247 |
| Model 1 — unadjusted | Q4 | 3.892 | 2.806–5.398 | <0.001 |
| Model 2 — primary parsimonious | Q1 | Reference | Reference | Reference |
| Model 2 — primary parsimonious | Q2 | 0.969 | 0.640–1.467 | 0.882 |
| Model 2 — primary parsimonious | Q3 | 1.170 | 0.787–1.737 | 0.438 |
| Model 2 — primary parsimonious | Q4 | 2.972 | 2.108–4.189 | <0.001 |
| Model 3 — extended sensitivity | Q1 | Reference | Reference | Reference |
| Model 3 — extended sensitivity | Q2 | 0.883 | 0.580–1.344 | 0.562 |
| Model 3 — extended sensitivity | Q3 | 1.016 | 0.682–1.515 | 0.936 |
| Model 3 — extended sensitivity | Q4 | 2.102 | 1.464–3.018 | <0.001 |

Model 1 was unadjusted. Model 2 was the primary parsimonious model adjusted for age, gender, BMI, SOFA score, type 2 diabetes, hypertension, pneumonia, septic shock, stroke, renal disease, malignant cancer, congestive heart failure, and glucocorticoid use. Model 3 was the extended sensitivity model including Model 2 covariates plus additional physiological and laboratory variables.

**Table S9. Sensitivity analyses using alternative outlier-handling strategies.**

| **Sensitivity strategy** | **Model** | **SHR quartile** | **HR** | **95% CI** | **P value** |
| --- | --- | --- | --- | --- | --- |
| Clinically implausible values removed | Model 1 | Q1 | Reference | Reference | Reference |
| Clinically implausible values removed | Model 1 | Q2 | 0.938 | 0.623–1.413 | 0.760 |
| Clinically implausible values removed | Model 1 | Q3 | 1.370 | 0.940–1.996 | 0.102 |
| Clinically implausible values removed | Model 1 | Q4 | 4.035 | 2.922–5.571 | <0.001 |
| Clinically implausible values removed | Model 2 | Q1 | Reference | Reference | Reference |
| Clinically implausible values removed | Model 2 | Q2 | 0.825 | 0.518–1.316 | 0.420 |
| Clinically implausible values removed | Model 2 | Q3 | 1.263 | 0.832–1.915 | 0.272 |
| Clinically implausible values removed | Model 2 | Q4 | 2.859 | 1.975–4.138 | <0.001 |
| Winsorization | Model 1 | Q1 | Reference | Reference | Reference |
| Winsorization | Model 1 | Q2 | 0.938 | 0.623–1.413 | 0.760 |
| Winsorization | Model 1 | Q3 | 1.370 | 0.940–1.996 | 0.102 |
| Winsorization | Model 1 | Q4 | 4.035 | 2.922–5.571 | <0.001 |
| Winsorization | Model 2 | Q1 | Reference | Reference | Reference |
| Winsorization | Model 2 | Q2 | 0.825 | 0.518–1.316 | 0.420 |
| Winsorization | Model 2 | Q3 | 1.263 | 0.832–1.915 | 0.272 |
| Winsorization | Model 2 | Q4 | 2.859 | 1.975–4.138 | <0.001 |

Clinically implausible values were removed in sensitivity strategy 1. Winsorization was used in sensitivity strategy 2 to limit the influence of extreme values while retaining clinically meaningful observations.

**Table S10. Sensitivity analyses comparing MissForest and MICE imputation.**

| **Imputation method** | **Model** | **Categories** | **HR** | **95% CI** | **P value** |
| --- | --- | --- | --- | --- | --- |
| MissForest | Model 1 | Q1 | Reference | Reference | Reference |
| MissForest | Model 1 | Q2 | 0.957 | 0.636–1.440 | 0.833 |
| MissForest | Model 1 | Q3 | 1.257 | 0.853–1.851 | 0.247 |
| MissForest | Model 1 | Q4 | 3.892 | 2.806–5.398 | <0.001 |
| MissForest | Model 2 | Q1 | Reference | Reference | Reference |
| MissForest | Model 2 | Q2 | 0.969 | 0.640–1.467 | 0.882 |
| MissForest | Model 2 | Q3 | 1.170 | 0.787–1.737 | 0.438 |
| MissForest | Model 2 | Q4 | 2.972 | 2.108–4.189 | <0.001 |
| MissForest | Model 3 | Q1 | Reference | Reference | Reference |
| MissForest | Model 3 | Q2 | 0.883 | 0.580–1.344 | 0.562 |
| MissForest | Model 3 | Q3 | 1.016 | 0.682–1.515 | 0.936 |
| MissForest | Model 3 | Q4 | 2.102 | 1.464–3.018 | <0.001 |
| MICE | Model 1 | Q1 | Reference | Reference | Reference |
| MICE | Model 1 | Q2 | 0.957 | 0.636–1.440 | 0.833 |
| MICE | Model 1 | Q3 | 1.257 | 0.853–1.851 | 0.247 |
| MICE | Model 1 | Q4 | 3.892 | 2.806–5.398 | <0.001 |
| MICE | Model 2 | Q1 | Reference | Reference | Reference |
| MICE | Model 2 | Q2 | 0.970 | 0.641–1.469 | 0.886 |
| MICE | Model 2 | Q3 | 1.171 | 0.788–1.739 | 0.435 |
| MICE | Model 2 | Q4 | 2.972 | 2.108–4.189 | <0.001 |
| MICE | Model 3 | Q1 | Reference | Reference | Reference |
| MICE | Model 3 | Q2 | 0.875 | 0.574–1.333 | 0.534 |
| MICE | Model 3 | Q3 | 0.992 | 0.666–1.479 | 0.969 |
| MICE | Model 3 | Q4 | 2.085 | 1.450–2.999 | <0.001 |

**Table S11. Predictive performance of SOFA, SHR, and SOFA + SHR for 28-day all-cause mortality.**

| **Model** | **C-index/AUC** | **AUC lower 95% CI** | **AUC upper 95% CI** | **Calibration intercept** | **Calibration slope** | **Brier score** |
| --- | --- | --- | --- | --- | --- | --- |
| SOFA | 0.602 | 0.565 | 0.639 | 0.000 | 1.000 | 0.106 |
| SHR | 0.690 | 0.655 | 0.725 | 0.000 | 1.000 | 0.100 |
| SOFA + SHR | 0.706 | 0.673 | 0.740 | 0.000 | 1.000 | 0.099 |

**Table S12. DeLong comparison of SOFA, SHR, and SOFA + SHR.**

| **Comparison** | **AUC model 1** | **AUC model 2** | **AUC difference** | **P value** |
| --- | --- | --- | --- | --- |
| SHR vs SOFA | 0.690 | 0.602 | 0.088 | <0.001 |
| SOFA + SHR vs SOFA | 0.706 | 0.602 | 0.105 | <0.001 |
| SOFA + SHR vs SHR | 0.706 | 0.690 | 0.017 | 0.101 |

**Table S13. Candidate machine-learning model grid.**

Candidate model

Lasso + StepCox [both]

survival-SVM

CoxBoost + survival-SVM

Ridge

Lasso + survival-SVM

SuperPC

CoxBoost + Ridge

Enet [alpha=0.1]

CoxBoost + Enet [alpha=0.1]

Enet [alpha=0.2]

Enet [alpha=0.3]

CoxBoost + Enet [alpha=0.3]

CoxBoost + Enet [alpha=0.2]

Enet [alpha=0.4]

CoxBoost + Enet [alpha=0.4]

Lasso + CoxBoost

Enet [alpha=0.5]

CoxBoost

CoxBoost + Enet [alpha=0.5]

Enet [alpha=0.6]

CoxBoost + Enet [alpha=0.6]

CoxBoost + Enet [alpha=0.7]

CoxBoost + Enet [alpha=0.8]

Enet [alpha=0.8]

Enet [alpha=0.9]

Lasso

Enet [alpha=0.7]

CoxBoost + Enet [alpha=0.9]

CoxBoost + Lasso

Lasso + plsRcox

CoxBoost + plsRcox

CoxBoost + StepCox [forward]

Lasso + StepCox [forward]

RSF + survival-SVM

CoxBoost + SuperPC

StepCox [forward]

plsRcox

RSF + Ridge

RSF + Enet [alpha=0.1]

Lasso + SuperPC

RSF + plsRcox

RSF + StepCox [forward]

RSF + Enet [alpha=0.2]

RSF + Enet [alpha=0.3]

RSF + Enet [alpha=0.6]

RSF + Lasso

RSF + Enet [alpha=0.7]

RSF + Enet [alpha=0.5]

RSF + CoxBoost

RSF + Enet [alpha=0.9]

RSF + Enet [alpha=0.4]

RSF + Enet [alpha=0.8]

RSF + StepCox [both]

RSF + StepCox [backward]

StepCox [both] + Ridge

StepCox [backward] + Ridge

StepCox [both] + plsRcox

StepCox [backward] + plsRcox

StepCox [both] + Enet [alpha=0.9]

StepCox [backward] + Enet [alpha=0.9]

StepCox [both] + Enet [alpha=0.1]

StepCox [backward] + Enet [alpha=0.1]

StepCox [both] + Enet [alpha=0.8]

StepCox [backward] + Enet [alpha=0.8]

StepCox [both] + Enet [alpha=0.2]

StepCox [backward] + Enet [alpha=0.2]

StepCox [both] + Lasso

StepCox [backward] + Lasso

StepCox [both] + Enet [alpha=0.6]

StepCox [backward] + Enet [alpha=0.6]

CoxBoost + GBM

StepCox [both] + Enet [alpha=0.7]

StepCox [backward] + Enet [alpha=0.7]

Lasso + StepCox [backward]

StepCox [both]

StepCox [backward]

CoxBoost + StepCox [both]

CoxBoost + StepCox [backward]

StepCox [both] + Enet [alpha=0.4]

StepCox [backward] + Enet [alpha=0.4]

StepCox [both] + Enet [alpha=0.3]

StepCox [backward] + Enet [alpha=0.3]

StepCox [both] + CoxBoost

StepCox [backward] + CoxBoost

StepCox [both] + Enet [alpha=0.5]

StepCox [backward] + Enet [alpha=0.5]

CoxBoost + RSF

RSF + SuperPC

RSF

Lasso + GBM

RSF + GBM

GBM

StepCox [both] + survival-SVM

StepCox [backward] + survival-SVM

Lasso + RSF

StepCox [both] + GBM

StepCox [backward] + GBM

StepCox [both] + RSF

StepCox [backward] + RSF

StepCox [both] + SuperPC

StepCox [backward] + SuperPC

A total of 101 candidate models or two-step model combinations were evaluated. For the final CoxBoost + SuperPC model, CoxBoost penalty was optimized with optimCoxBoostPenalty (start.penalty = 500), the optimal boosting step was 81, and the selected SuperPC threshold was 1.524. Detailed final hyperparameter settings are provided in Table S18.

**Table S14. Pairwise comparison of 28-day time-dependent AUCs between CoxBoost + SuperPC and top-performing candidate models.**

| **Comparison** | **AUC final model** | **AUC comparator** | **AUC difference** | **P value** |
| --- | --- | --- | --- | --- |
| CoxBoost + SuperPC vs Lasso + SuperPC | 0.845 | 0.845 | 0.000 | NA |
| CoxBoost + SuperPC vs SuperPC | 0.845 | 0.837 | 0.007 | 0.609 |
| CoxBoost + SuperPC vs CoxBoost + survivalSVM | 0.845 | 0.602 | 0.242 | <0.001 |
| CoxBoost + SuperPC vs Lasso + survivalSVM | 0.845 | 0.602 | 0.242 | <0.001 |

**Table S15. Pairwise DeLong tests between CoxBoost + SuperPC and top-performing candidate models for the binary 28-day endpoint.**

| **Comparison** | **AUC final model** | **AUC comparator** | **AUC difference** | **P value** |
| --- | --- | --- | --- | --- |
| CoxBoost + SuperPC vs Lasso + SuperPC | 0.847 | 0.847 | 0.000 | 1.000 |
| CoxBoost + SuperPC vs SuperPC | 0.847 | 0.837 | 0.010 | 0.475 |
| CoxBoost + SuperPC vs CoxBoost + survivalSVM | 0.847 | 0.593 | 0.254 | <0.001 |
| CoxBoost + SuperPC vs Lasso + survivalSVM | 0.847 | 0.593 | 0.254 | <0.001 |

**Table S16. Formal interaction testing between SHR and phenotype.**

| **Interaction analysis** | **P for interaction** |
| --- | --- |
| SHR ≥ 0.93 × Phenotype | 0.456 |
| Continuous SHR × Phenotype | 0.031 |

The dichotomized SHR × phenotype interaction was not statistically significant, whereas the continuous SHR × phenotype interaction suggested possible phenotype-related heterogeneity.

**Table S17. Internal validation of the final CoxBoost + SuperPC model using bootstrap optimism correction and an independent validation cohort.**

| **Analysis** | **Cohort** | **Metric** | **Value** | **Description** |
| --- | --- | --- | --- | --- |
| Apparent discrimination | Training cohort | Apparent C-index | 0.814 | Final CoxBoost + SuperPC model before optimism correction |
| Bootstrap optimism | Training cohort | Mean optimism | 0.011 | Mean difference between bootstrap-sample and original-cohort C-indices |
| Optimism-corrected performance | Training cohort | Optimism-corrected C-index | 0.803 | Apparent C-index minus mean optimism |
| Independent validation | Validation cohort | C-index | 0.826 | Final locked model applied to a cohort not used for feature selection, model training, hyperparameter tuning, or cutoff determination |

**Table S18. Hyperparameter settings and final optimal values of the CoxBoost + SuperPC model.**

| Model component | Procedure or hyperparameter | Candidate or fixed setting | Final selected value |
| --- | --- | --- | --- |
| CoxBoost | Penalty optimization | optimCoxBoostPenalty; initial penalty = 500 | Optimized penalty = 1000 |
| CoxBoost | Boosting-step selection | 10-fold cross-validation using cv.CoxBoost; maxstepno = 500; K = 10; type = "verweij" | Optimal boosting step = 81 |
| SuperPC | Model type | type = "survival" | type = "survival" |
| SuperPC | s0.perc | s0.perc = 0.5 | 0.5 |
| SuperPC | Threshold selection | 10-fold cross-validation; n.threshold = 20; n.fold = 10; n.components = 3; min.features = 5 | Final threshold = 1.524 |
| Final model | Retained features | Features retained after CoxBoost + SuperPC workflow | 18 features |
